# Mesenchymal–epithelial transition in lymph node metastases of oral squamous cell carcinoma is accompanied by ZEB1 expression

**DOI:** 10.1186/s12967-023-04102-w

**Published:** 2023-04-19

**Authors:** Kai Horny, Christoph Sproll, Lukas Peiffer, Frauke Furtmann, Patricia Gerhardt, Jan Gravemeyer, Nikolas H. Stoecklein, Ivelina Spassova, Jürgen C. Becker

**Affiliations:** 1 Translational Skin Cancer Research, German Cancer Consortium (DKTK), 45141 Essen, Germany; 2 German Cancer Research Center (DKFZ), 69120 Heidelberg, Germany; 3 Department of Oral- and Maxillofacial Surgery, Medical Faculty, University Hospital of the Heinrich-Heine-University, Düsseldorf, Germany; 4 Department of Dermatology, University Medicine Essen, 45141 Essen, Germany; 5 Department of General, Visceral and Pediatric Surgery, Medical Faculty, University Hospital of the Heinrich-Heine-University Düsseldorf, Düsseldorf, Germany

**Keywords:** Single cell RNA, Oral cavity, Squamous cell carcinoma, Epithelial–mesenchymal plasticity, EMT, MET, ZEB1, Heterogeneity, Partial EMT

## Abstract

**Background:**

Oral squamous cell carcinoma (OSCC), an HPV-negative head and neck cancer, frequently metastasizes to the regional lymph nodes but only occasionally beyond. Initial phases of metastasis are associated with an epithelial–mesenchymal transition (EMT), while the consolidation phase is associated with mesenchymal–epithelial transition (MET). This dynamic is referred to as epithelial–mesenchymal plasticity (EMP). While it is known that EMP is essential for cancer cell invasion and metastatic spread, less is known about the heterogeneity of EMP states and even less about the heterogeneity between primary and metastatic lesions.

**Methods:**

To assess both the heterogeneity of EMP states in OSCC cells and their effects on stromal cells, we performed single-cell RNA sequencing (scRNAseq) of 5 primary tumors, 9 matching metastatic and 5 tumor-free lymph nodes and re-analyzed publicly available scRNAseq data of 9 additional primary tumors. For examining the cell type composition, we performed bulk transcriptome sequencing. Protein expression of selected genes were confirmed by immunohistochemistry.

**Results:**

From the 23 OSCC lesions, the single cell transcriptomes of a total of 7263 carcinoma cells were available for in-depth analyses. We initially focused on one lesion to avoid confounding inter-patient heterogeneity and identified OSCC cells expressing genes characteristic of different epithelial and partial EMT stages. RNA velocity and the increase in inferred copy number variations indicated a progressive trajectory towards epithelial differentiation in this metastatic lesion, i.e., cells likely underwent MET. Extension to all samples revealed a less stringent but essentially similar pattern. Interestingly, MET cells show increased activity of the EMT-activator ZEB1. Immunohistochemistry confirmed that ZEB1 was co-expressed with the epithelial marker cornifin B in individual tumor cells. The lack of E-cadherin mRNA expression suggests this is a partial MET. Within the tumor microenvironment we found immunomodulating fibroblasts that were maintained in primary and metastatic OSCC.

**Conclusions:**

This study reveals that EMP enables different partial EMT and epithelial phenotypes of OSCC cells, which are endowed with capabilities essential for the different stages of the metastatic process, including maintenance of cellular integrity. During MET, ZEB1 appears to be functionally active, indicating a more complex role of ZEB1 than mere induction of EMT.

**Supplementary Information:**

The online version contains supplementary material available at 10.1186/s12967-023-04102-w.

## Background

Head and neck squamous cell carcinoma (HNSCC) is the sixth most common cancer worldwide, with 890,000 new cases and 450,000 deaths in 2018 [1]. The survival for HNSCC patients has improved modestly over the past decades; however, this improvement is partially attributable to the emergence of human papillomavirus (HPV)-associated HNSCC that has a better prognosis than HPV-negative tumors [1]. One of the HPV-negative HNSCC subtypes is oral cavity squamous cell carcinoma (OSCC) which is mainly associated with tobacco and alcohol abuse [1]. OSCCs are often diagnosed at an early stage owing to the patient’s self-identification of the mass lesion and symptoms. Still regional lymph node metastases are frequent and thus, surgical removal of primary tumor is accompanied by neck dissection and radiotherapy [2]. Given the morbidity associated with this combined intervention, there is a need to identify molecular biomarkers to predict the presence of lymph node metastases and to prognosticate survival.

In many epithelial tumors, invasion and metastasis becomes possible through an epithelial–mesenchymal transition (EMT), i.e., a reactivation of an embryonic developmental program in which cells acquire migratory and invasive properties [3]. In EMT, epigenetic, transcriptional, and post-translational changes cause epithelial cells to break down the strong homotypic cell–cell junctions and adopt a mesenchymal morphology [4]. EMT has also been shown to impact the characteristics of mesenchymal cells in the tumor stroma either by cell polarization or as a direct contributor to the cancer-associated fibroblast (CAF) population [5, 6]. Conversely, CAFs also modify the EMT status of tumor cells.

Importantly, EMT should not be understood as a clearly defined process, but rather as many dynamic and complex processes, which may vary depending on tumor entity, stage, and microenvironment [4, 7, 8]. Thus, expression of EMT-related genes and their regulating transcription factors is highly heterogeneous, even within one cancer entity, between patients, in different lesions from one patient, and between individual cancer cells within one lesion [4, 9]. Since it is a continuous, dynamic, and reversible process, cancer cells can adopt a multitude of intermediate or partial states, e.g., epithelial to more mesenchymal or partial EMT (pEMT) states [7, 10–13]. Therefore, it has recently been recommended that this EMT continuum should rather referred to as epithelial-mesenchymal plasticity (EMP) [4, 12].

Single-cell analyses are a powerful tool to capture the EMP-associated heterogeneity of cancer cells and their impact on stromal cells. However, to date most EMP-related single-cell studies are based on controlled in vitro and in vivo experiments [7, 8, 11, 14, 15]. Particularly in HNSCC only few studies scrutinized EMP within freshly isolated tumor samples [10, 16, 17]. Of particular note is the seminal work of Puram et al. in which 2215 malignant cells from 18 patients were characterized, revealing multiple pEMT states with high variability in EMP-related gene expression [10].

In the work presented here, we investigated the cellular heterogeneity of 5 primary, 9 regionally metastatic OSCC lesions and 5 tumor-free lymph nodes isolated from 7 patients using multiplexed single-cell RNA sequencing (scRNAseq). In addition, we re-analyzed a recently published series of scRNAseq data from primary HNSCC that included 9 OSCC tumors to put our observations on an even broader data base [17]. Our results not only confirm the EMP-associated heterogeneity of cancer cells in primary and metastatic OSCC, but also demonstrate that immunomodulating CAFs are preserved in primary and metastatic OSCC. Furthermore, we demonstrated a mesenchymal-epithelial transition (MET) of OSCC cells in established lymph node metastases. Surprisingly, we observed a high activity of the EMT-activator ZEB1 in metastatic OSCC cells with epithelial differentiation, which was confirmed by co-expression of ZEB1 and cornifin-B protein in individual tumor cells.

## Methods

### Tissue samples

From 7 OSCC patients treated at the Department of Oral and Maxillofacial Plastic Surgery of the University Hospital of Heinrich Heine University Düsseldorf, we examined a total of 19 tissue samples—5 primary tumors and 14 potentially affected lymph nodes—by histopathological examination and bulk and single cell RNA sequencing. Of the 14 lymph nodes, 9 represented lymph node metastases as indicated by detection of carcinoma cells in histopathological examination and scRNAseq. The clinical details are provided in Additional file 10: Table S1. Due to the large size of the excised lesion, we were able to analyze two different areas of the primary tumors of patients #6 and #7 as separate samples to better capture any heterogeneity that may exist; these samples are designated #6.1 and #6.4 as well as #7.1 and #7.4, respectively.

### Histology and immunohistochemistry (IHC)

Hematoxylin and Eosin (H&E) and IHC were performed on 4 µm formalin-fixed, paraffin-embedded (FFPE) sections. H&E staining was performed using standard protocols (Additional file 1: Fig. S1). Whole-slide imaging was performed using Zeiss Axioscan 7 and 10 × magnification (Carl Zeiss Microscopy Deutschland GmbH, Oberkochen, Germany).

IHC using the rabbit polyclonal antibodies anti-SPRR1B (Cat. No.: SAB1301567-400UL, Sigma Aldrich, Darmstadt, Germany) and anti-ZEB1 (Cat. No. HPA027524-25UL, Sigma Aldrich) was performed as previously described [18]. Briefly, after sections were deparaffinized for 60 min at 60 °C and rehydrated, sections were incubated for 15 min in an inverter microwave oven with antigen retrieval buffer pH 9 for anti-SPRR1B and pH 6 for anti-ZEB1. After 3 × 2 min washes with Tris-buffered saline with 0.1% Tween (TBST) sections were incubated for 8 min with 3% peroxidase. Following an additional washing step, slides were incubated for 30 min with 3% bovine serum albumin (BSA) in TBST. For single stainings, sections were incubated at room temperature for 1 h with anti-SPRR1B at a dilution of 1:600, or overnight with anti-ZEB1 at a dilution of 1:500 dilution. Afterwards, the secondary anti-rabbit HRP Polymer was applied for 30 min, followed by 1:20 diluted 3,3′-Diaminobenzidin (DAB) for 10 min and 1:10 diluted hematoxylin for 3 min. Samples were washed with TBST in between incubations and, with tap water for 3 min before fixation. For multiplexed antigen detection, the OpalTM chemistry system (Akoya Biosciences, Marlborough, MA, USA, Cat. No.: OP7TL4001KT) was used according to the manufacturer’s description. Briefly, after deparaffinization and fixation, we processed the sections for 15 min with retrieval buffers in an inverter microwave oven. Then, we incubated them with antibody diluent for 10 min at room temperature, followed by incubation with the anti-SPRR1B antibody for 30 min. Next, Opal Polymer horseradish peroxidase (HRP) secondary antibody solution with the respective chromogen was applied for 10 min, antibodies were removed by microwave treatment and the staining with anti-ZEB1 antibody was performed. Finally, slides were incubated with 4′,6-diamidino-2-phenylindole (DAPI) for 5 min.

### Single-cell RNA sequencing

Samples were processed immediately after surgery and temporarily stored for transport at 4 °C in tissue storage solution before processing (Miltenyi Biotec, Bergisch Gladbach, Germany). Briefly, samples were dissociated into single-cell suspensions using the gentleMACS Dissociator (Cat. No. 130-093-235, Miltenyi Biotec, Bergisch Gladbach, Germany) with program “h_tumor_01”, followed by 2 × program “h_tumor_02” in 4.7 ml RPMI 1640 (Cat. No. P04-16500, PAN-Biotech) and an enzyme mix consisting of 200 µl Enzyme H, 100 µl Enzyme R and 25 µl Enzyme A (Cat. No. 130-095-929 Miltenyi Biotec). Afterwards, single-cell suspensions were reconstituted and washed thrice with 0.05% BSA phosphate-buffered saline (PBS) and filtered through a 100 µl cell strainer.

In cases multiple samples of a single patient had to be analyzed (Additional file 10: Table S1), antibody hashing for multiplexing of samples was performed according to manufacturer’s protocol. Briefly, 1 µg of the respective TotalSeq anti-human hashtag antibody was used to incubate a maximum of ca. 2 million cells for 30 min at 4 °C (Cat. No. 394601, 394603, 394605 and 394661, 394663, 394665, respectively, Biolegend, San Diego, CA, USA). After 3 washes with PBS with 0.05% BSA, the respective cell suspensions were mixed prior to single-cell RNA library preparation. In short, both unhashed and hashed single-cell suspensions were barcoded and processed with the microfluidic system of 10 × Genomics Chromium v2.0 platform as described in the manufacturer’s protocols (10 × Genomics, Leiden, Netherlands). Due to a change of system, both the 3′ technology including Chromium Single Cell 3′ Library & Gel Bead Kit version 2 (Cat. No. 120237), Chromium Single Cell A Chip Kit (Cat. No. 120236) and Chromium i7 Multiplex Kit (Cat. No. 120262), as well as the 5′ technology including Chromium Single Cell 5′ Library & Gel Bead Kits version 2 (Cat. No. 1000263), Chromium Next GEM Chip K Single Cell Kit (Cat. No. 1000286) and Dual Index Kit TT set A (Cat. No. 1000215) were used; for library construction the Chromium Single Cell 3′/5′ Library Construction Kit (Cat. No. 1000020) was applied. After library preparation, the library from patient #1 was sequenced with an Illumina HiSeq 4000 (Illumina, Berlin, Germany) at the DKFZ Genomics and Proteomics Core Facility in Heidelberg and all other libraries were sequenced on an Illumina NovaSeq 6000 (Illumina, Berlin, Germany) in three runs (Run 1: patient #2, #4, #5; Run 2: patient #3, Run 3: patient #6 and #7) at the West German Genome Center in Cologne.

### Bulk transcriptome analysis

The bulk transcriptome was analyzed using a quantitative nuclease protection assay from the HTG Transcriptome Panel (HTP) according to the manufacturer’s protocol (Cat. No. HTG-001-224, HTG Molecular Diagnostics, Tucson, AZ, USA). Briefly, the tumor areas were macro-dissected as depicted in Additional file 1: Fig. S1 from 4 µm FFPE sections and subjected to Proteinase K and DNase digestion. Next, the quantitative nuclease protection assay was performed using the HTG EdgeSeq Processors before adapters and sample tags were added during PCR amplification. The resulting libraries were sequenced using an Illumina NextSeq 500/550 High Output Kit v2.5 (75 cycles) (Cat. No. 20024906, Illumina, Berlin, Germany).

The resulting FASTQ files were processed towards a gene expression count matrix using the HTG EdgeSeq Reveal Software version 4.0.1. Quality Control, normalization, and principal component analysis (PCA) were performed using R version 4.0.5. Sample 5 failed QC due to low number of sequenced reads and was removed from the analysis. Deconvolution was performed with web application of CIBERSORTx (https://cibersortx.stanford.edu/) using a signature matrix derived from the gene expression count matrix of combined scRNAseq data of the samples analyzed with HTP [19]. We filtered for genes that are expressed less than 5% within the given tumor phenotypes and randomly included only 75% of T and B cells for better performance. The resulting signature matrix was used for imputing cellular fractions from the counts-per-million normalized HTP data without any batch correction or quantile normalization and 500 permutations.

### Bioinformatic analysis of scRNAseq data

#### Preprocessing

Processing from FASTQ files towards the unfiltered count matrix (barcodes × genes) was performed using Cellranger Software Suite version 3.1.0 and the human reference genome build GRCh38, downloaded from 10 × Genomics in version 3.0.0.

Cells were identified by evaluating quality criteria inspired by Luecken et al*.* (see Additional file 11) [20]. Cells were defined by having more than 500 unique molecular identifiers (UMIs), less than 10% mitochondrial gene expression and additionally for patient 3 and 5 having more than 30 housekeeper genes expressed. The filtered count matrices (cells × genes) were further processed using Seurat version 4.0.1 and R version 4.0.5 [21]. Demultiplexing of hashed libraries was performed choosing manual threshold of hashtag oligo (HTO) expression based on quality assessments described in Additional file 11. We removed doublets that were identified by demultiplexing of HTO expression matrices from all analyses.

#### Normalization and dimensionality reduction

When performing analysis of a specific set of cells, e.g., only tumor cells or only cells of a specific patient, the respective set of cells was normalized using the SCtransform algorithm and the 3000 most variable genes were selected for PCA [22]. During normalization, we regressed for cell cycle scores and percentage of mitochondrial gene expression. Cell cycle scores and phases were determined in Seurat using log-normalized RNA counts and S and G2M-Phase genes defined by Tirosh et al. [23]. When generating a uniform manifold approximation and projection (UMAP) without patient-specific batch effect, we used corrected PCs derived with the harmony R package version 0.1.0 [54]. Based on the variance explained by each PC and the respective ranked elbow plot we choose an appropriate number of PCs for UMAP visualization and SNN clustering as implemented in Seurat. For deriving patient-specific clusters for calculating the intratumoral cosine similarity, we used the same resolution parameter for comparability. UMAPs colored by specific gene expression were ordered by expression values.

#### Tumor cell identification and phenotyping

We annotated all cell types and identified tumor and fibroblast phenotypes by using a combination of methods: SNN clustering, differential gene expression, gene set enrichment analysis (GSEA), expression of literature-based marker genes and automated reference-based annotation with SingleR using the Monaco bulk-RNA Immune dataset (Additional file 1: Fig. S2) [24, 25]. Automated, reference-based annotation with SingleR version 1.4.1 was run on SNN clusters with a resolution of 100, yielding extremely small clusters including only few cells but increasing performance. Further, we excluded cells with ambiguous cell type marker expression. For example, within the tumor cells of the lymph node metastasis from patient 1, we observed few cells expressing genes typical for fibroblasts and DCs that were subsequently excluded from tumor-specific analysis. Similarly, we removed T cells, B cells, mast cells, fibroblasts, muscle cells, melanocytes, and other immune cells from the tumor cell subsets, as well as T cells highly expressing CD3 genes from the fibroblast subset. Malignant cells were first identified by high epithelial gene expression, e.g., high cytokeratin expression, and inferred CNVs (Additional file 2: Fig. S2 C–E). CNVs were inferred using the R package inferCNV version 1.6.0 with the not normalized, filtered count matrix including all cells as input and algorithm run in “samples” mode. Inferred CNVs of mitochondrial genes were excluded. Differential gene expression was performed by calculating the log2 foldchanges between one cluster and all other cells from the subset based on log-normalized data using NormalizeData function and a scale factor of 10,000. We filtered for genes with log2 foldchange greater than 0.25 and a minimum percentual expression of at least 10% within the cluster or all other cells. For calculating the cosine similarity, we did not filter the log2 foldchanges. GSEA was performed using the “fgsea” R package version 1.16.0, log2 foldchanges from differential gene expression and gene ontology biological processes (GO:BP,C5 v7.1) as well as hallmark gene sets (H, v7.1) downloaded from MSigDB database [26–28]. Gene sets were included if they had at least 15 genes or at maximum 500 genes within the gene set using 10,000 permutations.

For deriving epithelial differentiating and pEMT gene signatures of patient 1 we calculated foldchanges between the “epi” and “pEMT” cluster and included genes with log2 foldchanges greater or lesser than 1, respectively, and with at least 10% of either epi or pEMT cells expressing that gene. Gene set variation analysis (GSVA) was performed using the R package GSVA version 1.38.2 with default settings, i.e., gaussian kernel [29]. As input, EMTome signatures, the pEMT and epithelial differentiation 1 and 2 signature from Puram et al. [10] and the three EMT and the epithelial senescence signatures from Kinker et al*.* were used [14].

Trajectories were inferred using SlingShot version 1.8.0 with malignant cell clusters as shown in Fig. 2A of patient 1 and the first 20 PCs [30]. RNA velocity was inferred using the VeloCyto python and R package (version 0.6) [31]. Creation of the loom file was done using default options and gene annotations as used for Cellranger processing. Genes were filtered based on the minimum average expression magnitude with a threshold of 0.05 for spliced and 0.02 for unspliced reads. Velocity estimates were calculated using the inverse correlation coefficient of the PC embedding correlation matrix as distance, the top/bottom 2% quantiles for gamma fit, 50 neighboring cells projecting 1 deltaT into the future and projected on the UMAP using 200 neighbors and 30 grid points.

Transcription factor activity was inferred using the VIPER algorithm (version 1.24.0) and regulons from the DoRothEA database (version 1.2.2) [32–34]. Hierarchical clustering in the heatmaps was performed using the Euclidean distance and ward.D2 method unless otherwise noted. Visualization was performed using ggplot2 version 3.3.3 and ComplexHeatmap version 2.6.2 [35, 36].

### Reanalysis of HNSCC dataset from Kürten et al.

From the publicly available scRNAseq data set on primary HNSCC tumors we downloaded the FASTQ files of CD45-negative and HPV-negative libraries from the sequencing read archive (SRA) under accession ID SRP301444 [17]. The data sets were analyzed the same as described above. HPV-negative samples were chosen for comparability to the OSCC dataset. However, the HN07 tumor originated from the larynx, while all other samples originated from the oral cavity. We adjusted the cell identification thresholds based on our evaluation criteria pooled for all libraries: cells were defined by having more than 175 genes expressed, less than 10% mitochondrial gene expression and more than 60 housekeeper genes expressed (see Additional file 11).

## Results

### Single-cell gene expression signatures of tumor cells from a single metastasis show several predominant, but not necessarily exclusive, functional phenotypes

**Fig. 1:**
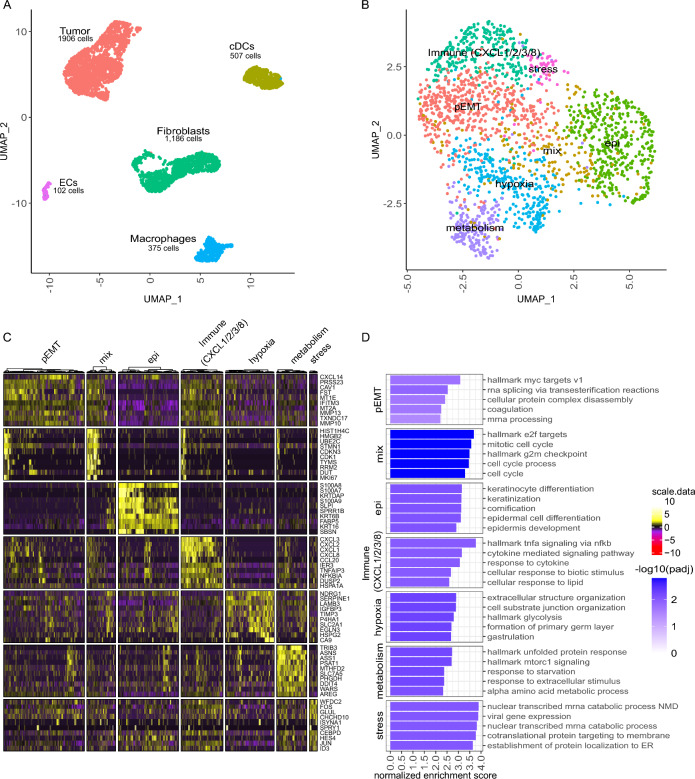
Single-cell gene expression signatures in OSCC cells from a single metastasis reveal predominant functional phenotypes. **A** UMAP based on scRNAseq data of 4076 cells isolated from a metachronous lymph node metastasis. Cells are annotated and summarized according to the presumed cell type. **B** UMAP of 1906 OSCC cells depicted in **A**. Cells are annotated according to predominant functional phenotype. **C** Heatmap for scaled, log-normalized gene expression of tumor cells (columns) split by respective phenotype and the top 10 differentially expressed genes (DEGs) (rows) of the respective phenotype against all other tumor cells. DEGs are sorted from highest to lowest log2 foldchange. Row sections are ordered like column sections. **D** Top 5 enriched gene sets from log2 foldchanges of respective tumor phenotypes by normalized enrichment scores (x-axis). Gene sets of respective phenotypes are sorted from highest to lowest enrichment. Bars are colored by the negative decadic logarithm of the Benjamini–Hochberg adjusted p-value (padj). DCs: dendritic cells. ECs: endothelial cells

To avoid inter-patient heterogeneity as a confounding factor, we first focused on the analysis of a single OSCC metastasis to develop hypotheses which would be subsequently tested in the entire cohort. For this, we chose a metachronous lymph node metastasis that was removed one year after the primary tumor, because we assumed that consolidation processes are particularly pronounced in this longer existing metastasis. Multiplexed scRNAseq recovered 4121 cells that could be assigned to the following cell types: 1906 (46.8%) tumor cells, 1186 (29.1%) fibroblasts, 507 (12.4%) dendritic cells (DCs), 375 (9.2%) macrophages and 102 (2.5%) endothelial cells (Fig. 1A). The absence of T or B cells was in line with the histology showing completely disrupted lymph node structures and only occasional tumor-infiltrating lymphocytes (Additional file 1: Fig. S1). OSCC cells were identified both by the presence of copy number variations (CNVs) inferred from scRNAseq data as well as the expression of epithelial markers including *S100A2,* cytokeratins (*KRT5*, *KRT14*, *KRT17)* and stratifin (*SFN*) (Additional file 2: Fig. S2C–E).

Detailed phenotyping of the cancer cells identified several clusters to which we could assign predominant functional phenotypes that differ in their EMT state, immunomodulatory capacity, as well as their response to hypoxia, stress, and metabolic constraints (Fig. 1B-D). However, the predominance of a functional phenotype does not exclude additional traits. Specifically, 515 cells exhibited a pEMT phenotype characterized by expression of a mixture of epithelial and mesenchymal genes such as matrix metallopeptidases (*MMPs*), caveolin-1 (*CAV1*) and C–X–C motif chemokine ligand 14 (*CXCL14*). Those genes were previously described in pEMT signatures and are enriched in the EMT hallmark gene set from the molecular signatures database (MSigDB) (Additional file 3: Fig. S3A) [10, 27]. In contrast, 385 cells showed higher expression of genes associated with epithelial differentiation such as *S100A7/A8/A9,* the keratinocyte envelope protein cornifin-B (*SPRR1B*), and specific cytokeratins (e.g., *KRT6B* and *KRT16*). The EMP-related gene expression patterns correlate with established signatures from the EMTome database (Additional file 3: Fig. S3B–E) [9]. Interestingly, 184 cells from both EMP-phenotypes were present in a mixed cluster characterized by the high expression of cell-cycle related genes (despite the fact that we applied cell cycle regression).

With respect to cell clusters whose gene expression was not predominately associated with EMP, 268 cells exhibited an immune-regulatory phenotype enriched for genes associated with cytokine-mediated responses and higher expression of the chemokines *CXCL1/2/3/8* and *CCL20*. For 554 cells, the gene expression pattern suggests adaptation to environmental factors. Specifically, 51 cells had higher expression of transcription factors *FOS* and *JUN,* suggesting a stress response, and 310 cells can be assumed to respond to hypoxic conditions in the tumor based on the higher expression of *NDRG1* and *EGLN3*, which are both regulated by oxygen levels [37, 38]: *NDRG1* regulates stress response and p53-mediated caspase activation [37] and *EGLN3* has an important role in regulation of hypoxia-inducible factor 1 alpha (HIF1α) through prolyl hydroxylation [38]. 193 cells expressed genes associated with amino acid metabolism, starvation response and mTORC1 signaling, i.e., a regulator of mitochondrial metabolism [39]. The upregulated genes *ASNS*, *PSAT1* and *PHGDH* integrate the metabolites of serine and glycine metabolism into glycolysis and therefore fuel glycolysis with amino acids [40]; hence, these cells appear to be adapted to low-glucose conditions.

### OSCC cells in lymph node metastases undergo mesenchymal-epithelial transition

**Fig. 2:**
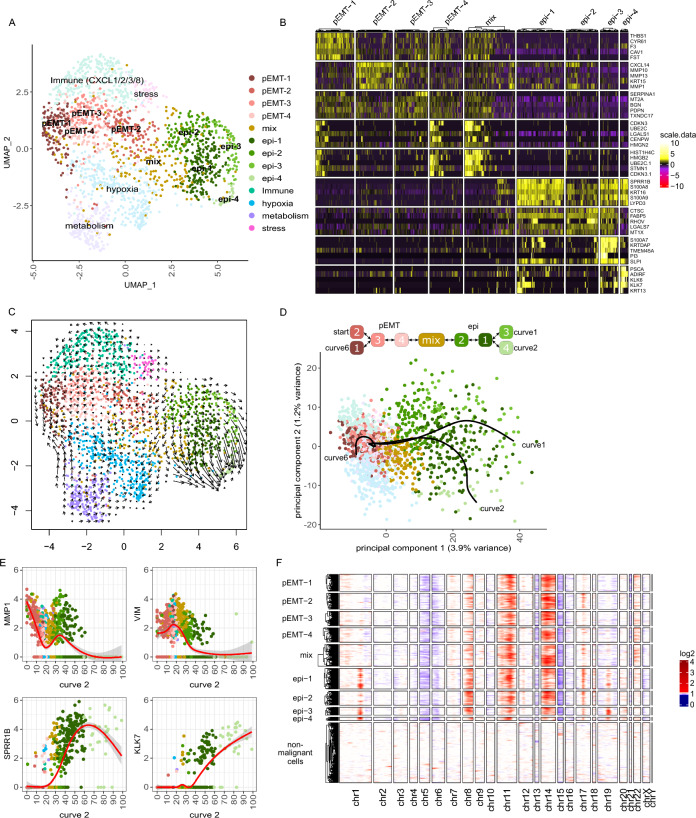
A progressive epithelial differentiation, but no strong uniform direction of development in pEMT clusters. **A** UMAP of 1906 OSCC cells annotated based on SNN clustering, defining 4 pEMT (pEMT-1 to 4), 4 epithelial differentiated (epi-1 to 4) and one mixed (mix) cluster; clusters are numbered by size. **B** Heatmap for scaled, log-normalized gene expression in EMP-associated tumor cell phenotypes (columns) split by EMP cluster and their top 5 DEGs (rows) against all other EMP-related tumor cell phenotypes. DEGs are sorted from highest to lowest log2 foldchange. Row sections are ordered like column sections. **C** Projection of RNA velocity on the UMAP depicted in A. Arrows indicate the extrapolated direction of development; arrow length indicates strength of future development. **D** First two principal components of OSCC cells with the three EMP-related principal curves that are derived from trajectory inference. Graph on top visualizes the relationship between EMP clusters described by the three principal curves forming a branching trajectory. **E** Log-normalized expression (y-axis) of *MMP1*, *VIM*, *SPRR1B* and *KLK7* across pseudotime values (x-axis) of curve 2, color-coded by clusters. Red lines indicate smoothed expression values over the trajectory generated with a general additive model; 95% confidence intervals are shaded gray. **F** Inferred CNVs across EMP-related tumor cells (rows) for all chromosomes (columns). Red indicates copy number gains, white diploid copy number and blue copy number loss. Columns show genes categorized in chromosomes and ordered by genome position; hence the size of the chromosome reflects the number of detected genes and not its nucleotide length. Mitochondrial genes were excluded

We next focused on possible dynamics within the predominantly EMP-related cancer cell clusters using a higher resolved shared-nearest neighbor (SNN) clustering. Based on this, we defined 4 pEMT clusters (pEMT-1 to 4), 4 clusters of more epithelial differentiated cells (epi-1 to 4) and one cluster with mixed phenotypes (mix) (Fig. 2A). pEMT-1 is enriched for genes involved in coagulation such as *THBS1*, *CYR61* and *F3,* and may play a role in angiogenesis (Fig. 2B, Additional file 4: Fig. S4A). pEMT-2 and 3 both showed higher expression of extracellular matrix (ECM) remodeling genes, but in addition pEMT-2 was characterized by higher expression of cytokeratin *KRT15* and chemokine *CXCL14* while pEMT-3 showed higher expression of the serine protease inhibitor *SERPINA1* and podoplanin (*PDPN*) which mediates efficient ECM degradation by controlling invadopodia [41]. Of the more epithelial differentiated cell clusters, epi-2’s expression profile is closest to the pEMT cluster, having higher expression of *MMP1* and lower expression of *SPRR1B* and *S100A8/A9* than epi-1, epi-3, or epi-4 (Fig. 2B, E). Epi-3 showed increased expression of *S100A7* and *KRTDAP*, whereas epi-4 showed higher expression of kallikreins (*KLK6/7*), prostate stem cell antigen (*PSCA*) and adipogenesis regulatory factor (*ADIRF*)*.* Both *ADIRF* and *PSCA* play a role in prostate cancer and *PSCA* is also reported as highly expressed in mucosal tissue, but less in HNSCC [42–44].

To gain a better understanding on the gene expression dynamics in this metastasis, we estimated RNA velocity, which predicts the short-term future development in gene expression of individual cells using the ratio of spliced and non-spliced mRNA counts (Fig. 2C) [31]. This analysis revealed that epithelial differentiated cells were strongly developing towards cluster epi-4, while most other cells show more or less random patterns of developmental directions, hence could not be interpreted. Tracking the developmental pathway within the metastasis by trajectory analysis across all EMP-related clusters revealed a major developmental axis between pEMT and epithelial differentiated cells that is diversifying within each end (Fig. 2D, E, Additional file 4: Fig. S4B). To confirm that the found progressive epithelial differentiation of the metastatic cells represents an MET, we inferred CNVs from the scRNAseq data. This demonstrated an increased number of copy number gains on chromosome 1, 8, 17, and 19 within epithelial differentiated cell clusters epi-1, epi-3, and epi-4 (Fig. 2F, Additional file 4: Fig. S4C). It should be noted, however, that CNVs on chromosomes 1 and 17 are associated with upregulated epithelial genes in close genomic proximity; thus, these two copy number gains may not represent true genomic CNVs, but rather reflect the high expression of these genes in epithelial differentiated cells (Additional file 4: Fig. S4D, E).

### Intra-tumoral heterogeneity of OSCC is driven by EMP

**Fig. 3:**
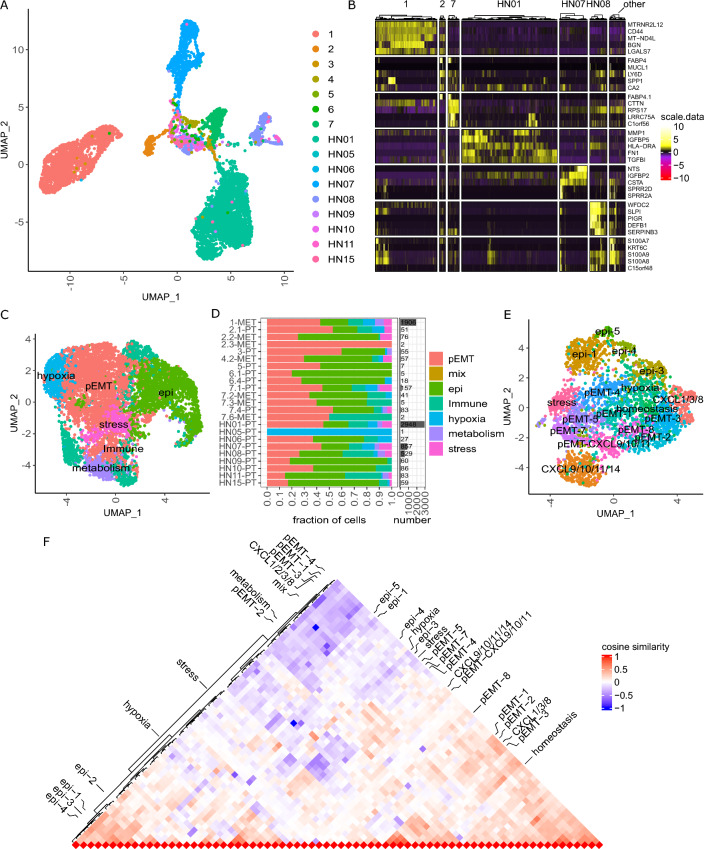
Intra-tumoral heterogeneity of OSCC is driven by EMP. **A** UMAP based on scRNAseq data of 7263 cancer cells from 16 different patients annotated by patient. **B** Heatmap for scaled, log-normalized gene expression of tumor cells split by patients and their top 5 DEGs (rows) against all other tumor cells. All patients with less than 100 cells are summarized in the ‘other’ column. DEGs are sorted from highest to lowest log2 foldchange. Row sections are ordered like column sections. **C** UMAP based on scRNAseq data depicted in A with PCs corrected for patient-specific effects using harmony. Cells are annotated according to their predominant phenotype. **D** Relative distribution of tumor cell phenotypes (left) and cancer cell abundance (right) across patients. The label on the y-axis shows the sample identification and tumor localization (primary tumor [PT] or lymph node metastasis [MET]). **E** UMAP based on scRNAseq data of 2948 OSCC cells from patient HN01. Cells were annotated based on SNN clustering and the predominant phenotype. **F** Triangle heatmap of cosine similarity comparing the intratumoral heterogeneity across all patients. Cosine similarity is calculated between log2 fold changes from patient-specific clusters against all other tumor cells within the respective patient. Left side annotated are patient-specific clusters from patient #1 depicted in Fig. 2A and right side from patient HN01 depicted in **E**. We included only patients with more than 50 tumor cells

To test whether our observation that OSCC cells in one lymph node metastasis undergo a mesenchymal-epithelial transition is generally valid, we extended our analyses by adding 5 primary tumors and 8 matched lymph node metastases from 6 patients (Additional file 10: Table S1). The patients presented with a history of tobacco smoking and alcohol abuse except the female patients #4 and #5, both of whom, however, also lack HPV positivity. From the publicly available scRNAseq data set on primary HNSCC tumors published by Kürten et al. [17] we chose to include the 9 HPV-negative primary tumors in our analysis, of which all but one originated from the oral cavity (HN07 originated from the larynx). In total, we analyzed 7263 cancer cells from 16 different patients (Fig. 3A). Importantly, the frequency of cancer cells was unevenly distributed across samples, which could not be explained by differences in tumor cell content across samples as determined for our cohort by histopathology (Additional file 1: Fig. S1). The stability of epithelial tumor cell assemblies, which may not be sufficiently broken up by dissociation protocols, likely interfered with the generation of OSCC single-cell suspensions (Additional file 5: Fig. S5A). In addition, as expected from the inter-patient heterogeneity, cancer cells were clustered based on their gene expression by patient rather than functional phenotype (Fig. 3A, B). Thus, we accounted for the patient-specific effects with batch-corrected principal components (PCs) using the harmony R package which indeed resulted in a clustering by functional phenotypes [45] (Fig. 3C, Additional file 5: Fig. S5B–D). For annotation of the phenotype of the clusters we were guided by the gene signatures previously identified in the indicator sample, but also found several additional, predominantly immunoregulatory phenotypes. EMP-related phenotypes were present in all but one tumor sample with only one EMP cell (Fig. 3D). To compare the EMP-related intrapatient heterogeneity of all analyzed tumor cells between patients, we performed differential gene expression within each patient and calculated the similarities between the resulting clusters of all patients. We first considered each patient individually and performed clustering, annotation, inferCNV and differential expression analysis. As exemplified for the primary OSCC of patient HN01, tumor cell clusters had the same inferred CNVs and were annotated based on their phenotype again using the indicator sample as a guide (Fig. 3E, Additional file 5: Fig. S5E, F). The cosine similarity between patient-specific clusters demonstrated that within each patient, the heterogeneity in EMP is most prominent and epithelial differentiated phenotypes are profoundly different from most other clusters, especially pEMT (Fig. 3F, Additional file 5: Fig. S5G). Indeed, all pEMT phenotypes are very similar to each other and show a large overlap of the gene expression patterns with predominantly immune- and metabolic-related clusters. In epithelial differentiated cells, the most upregulated genes are *S100A8* and *S100A9*, encoding calprotectin, and *SPRR1B*, which are all members of the epidermal differentiation complex [46]. Of note, hypoxia- and stress-related heterogeneity is similar between patients suggesting a reactive response rather than an aspect of tumor evolution.

### EMT-related transcription factor ZEB1 is highly active in metastatic epithelial differentiated OSCC cells

**Fig. 4:**
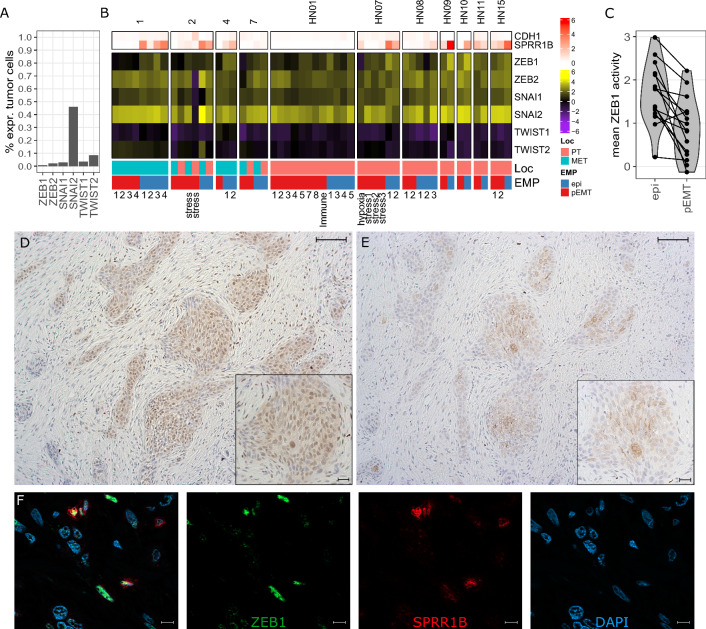
ZEB1 is highly active in metastatic epithelial differentiated OSCC cells. **A** Percentage of tumor cells with detectable mRNA expression from scRNAseq (more than one UMI) encoding the indicted EMP-related transcription factors. **B** Mean inferred activity based on the target genes of the indicated transcription factors across tumor phenotypes from EMP-related patient-specific clusters. On top the log-normalized expression of *CDH1* and cornifin-B (*SPRR1B*) is shown, on bottom the localization (primary tumor [PT] or lymph node metastasis [MET]) and respective EMP phenotype of the cluster. **C** Mean activity of ZEB1 for epithelial differentiated and pEMT clusters of each patient, respectively. Connecting lines show dots belonging to the same patient. ZEB1 (**D**) and cornifin-B (**E**) protein expression detected in serial sections by IHC of the primary tumor from patient #2; comparable areas are depicted. Scale bars equal 200 µm in overview and 100 µm in zoomed image. **E** Colocalized expression of ZEB1 (green) and cornifin-B (red) detected by double staining in the lymph node metastasis of patient #1. Nuclei are stained in blue (DAPI), Scale bars equal 10 µm

The transcription factors ZEB1/2, TWIST1/2, Snail (*SNAI1*) and Slug (*SNAI2*) are key for regulation of EMP [7, 27]. While mRNA expression of *SNAI2* within single OSCC cells was reported by Puram et al*.*, the other transcription factors were not detected [10]. Here, we confirm this observation as we detected *SNAI2* mRNA in almost half of the OSCC cells, but none of the other transcription factors (Fig. 4A). However, detection of lowly expressed genes such as transcription factors by scRNAseq, especially in 10X genomics technology, becomes unreliable due to dropout effects [47]. Also, the activity of transcription factors is often not reflected by the dynamics of their mRNA expression alone, as their activity additionally depends on protein stability and posttranslational modifications; for example, the ZEB1 protein is more stable than Snail [48]. To circumvent this problem, we inferred the activity of these transcription factors based on the mRNA expression profile of their target genes using the algorithm VIPER with regulons defined by DoRothEA database [32–34]. Using this approach, we were able to detect high activities of ZEB1, ZEB2, Snail and Slug in OSCC cells of different patients with varying EMP phenotype (Fig. 4B). This shows that epithelial differentiation in OSCC metastases is associated with higher activity of the EMT activator ZEB1 (Fig. 4C). Since, on the one hand, this was unexpected and, on the other hand, the method used to derive transcription factor activities potentially overestimates the activity for transcriptional repressors, we addressed the plausibility of this observation (Additional file 6: Fig. S6). Consistent with the fact that one of the main functions of ZEB1 is the downregulation of E-cadherin [49] (*CDH1*), OSCC cells generally showed low expression of *CDH1* (Fig. 4B). Next, we investigated the expression of the ZEB1 protein by immunohistochemistry (IHC) in all 14 tumor lesions of our cohort. We observed nuclear ZEB1 expression in similar tumor areas as cytoplasmic cornifin-B expression, which served as a marker of epithelial differentiation (Fig. 4D, E). Consequently, we validated the co-expression of ZEB-1 and cornifin-B in the same cell by immunofluorescence double-staining (Fig. 4F). In line with our scRNAseq data, colocalization of both proteins was observed in a fraction of cancer cells in 9/14 (64%) samples and double positive cells were more frequently observed in lymph node metastases (7/9, 78%; Additional file 10: Table S1) compared to primary tumors (2/5, 40%).

### Immunomodulating CAFs are present in primary tumors and tumor-involved lymph nodes

**Fig. 5:**
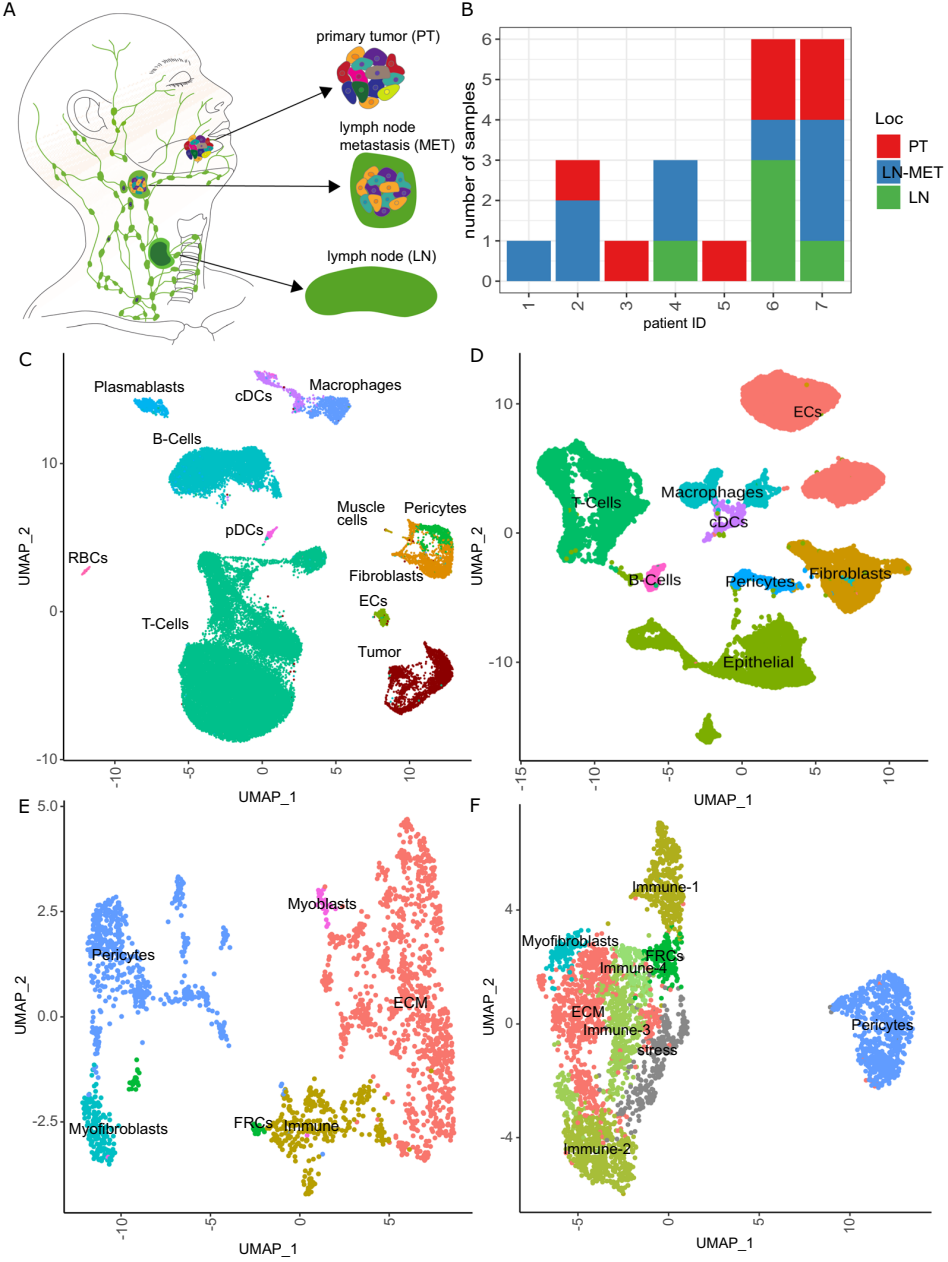
The OSCC microenvironment is composed of heterogenous fibroblasts from which immunomodulatory cells are present across the metastatic cascade. **A** Overview of types of analyzed OSCC samples and their localization within the head and neck area. **B** Number of samples across patients colored by their respective tissue origin; for patient #4, the primary tumor could not be analyzed due to incorrect specimen processing; for patients #6 and #7 two regions of the primary tumor were analyzed, denoted as sample #6.1 and #6.4 and #7.1 and #7.4, respectively. **C** UMAP of 41,284 cells based on OSCC scRNAseq data from our cohort and colored by cell type. **D** UMAP of 21,037 cells based on CD45-negative and HPV-negative primary HNSCC from Kürten et al*.* and colored by cell type. **E** UMAP of 1,595 fibroblasts and 551 pericytes from C colored by the respective phenotypes derived from shared-nearest neighbor clusters. **F** UMAP of 2,920 fibroblasts and 683 pericytes from D colored by the respective phenotypes derived from shared-nearest neighbor clusters

Next, we investigated the OSCC tumor microenvironment (TME) and derived its potential impact on metastatic dissemination. For this, we additionally analyzed the scRNAseq data of 5 tumor-free lymph nodes from patients #4, #6 and #7 (Fig. 5A, B). In this expanded cohort, most of the 41,284 cells were derived from the tumor-involved or tumor-free lymph nodes (34,599 cells, 84%), which as expected were predominantly immune cells, (35,856 cells, 87%, Fig. 5C, Additional file 7: Fig. S7A). The other non-malignant cells were fibroblasts (1595 cells, 4%), pericytes (551 cells, 1%), endothelial cells (399 cells, 1%) and muscle cells (55 cells, 0.1%). In comparison, due to the negative selection of CD45 + leukocytes, the data set of Kürten et al. shows a higher proportion of stromal cells, including endothelial cells (5972 cells, 28%), fibroblasts (3067 cells, 15%) and pericytes (673 cells, 3%, Fig. 5D, Additional file 7 Fig. S7B). Quantification of cell type composition in scRNAseq datasets is difficult to interpret because of technical biases in sample preparation (e.g., larger and stiffer cell types are generally underrepresented) that results in cell number and patient-specific differences (Additional file 7: Fig. S7C, D). Hence, we examined the bulk transcriptome and deconvoluted the respective cell types for our samples, revealing higher tumor and stroma cell content compared to cell type proportions derived from scRNAseq data (Additional file 8: Fig. S8A). Still, the tumor-free lymph nodes contained a high number of lymphocytes, whereas the metastatic samples had a composition similar to primary tumors despite the relevant differences between samples (Additional file 8: Fig. S8A, B).

Since the EMP status of tumor cells affects the properties of CAFs and vice versa, we focused on the transcriptional phenotypes of CAFs. Three main phenotypes were identified within our dataset: ECM-producing and -modifying fibroblasts (1071 cells, 67%), immunomodulating fibroblasts (311 cells, 19%) and contractile myofibroblasts (144 cells, 9%, Fig. 5E, Additional file 9: Fig. S9A–D). Additionally, there was a small population of fibroblast reticular cells (FRCs, 41 cells, 3%) and myoblasts (28 cells, 2%). These phenotypes were also present in the Kürten et al*.* dataset and due to the higher number of available cells, we were also able to differentiate the immunomodulating fibroblasts into further subtypes varying in their expression of chemokines and cytokines and to identify a fibroblast population associated with cell stress (Fig. 5F, Additional file 9: Fig. S9E–H).

The ECM-producing and -modifying phenotype is characterized by higher expression of MMPs, collagens (i.e., I, III, V and VI) and is enriched for gene sets related to formation and organization of the ECM (Additional file 9: Fig. S9A–C, E–G). Cells with contractile functions include pericytes identified by expression of the regulator of G-protein signaling 5 (*RGS5*), myofibroblasts identified by cytoskeleton genes such as alpha smooth muscle actin 2 (*ACTA2*), actin gamma smooth muscle 2 (*ACTG2*) and myosin heavy chain 11 (*MYH11*), and myoblasts identified by desmin (*DES*), chordin like 2 (*CHRDL2*) and transcription factors associated to myogenic differentiation (*MYF5/6*, Additional file 9: Fig. S9A, D, E, H). Moreover, myofibroblasts have enriched gene sets related to muscle contraction and similar to pericytes only express collagens IV and XVIII. Stress-associated cells express heat shock proteins (HSPA’s), AP-1 related genes *JUN* and *FOS* and also ECM-producing genes, indicating they are ECM-producing fibroblasts impregnated with a transcriptional stress response signature as the predominant phenotype (Additional file 9: Fig. S9G). Immunomodulating fibroblasts exhibit higher expression of chemokines such as *CXCL12* or *CXCL14*, cytokines such as Interleukin 6 (*IL6*), complement factors such as *C3* and *CFD,* and phospholipases such as *PLA2G2A* and *APOD,* with most enriched gene sets being related to immune response mechanisms (Additional file 9: Fig. S9A, D, E, H). Hence, these cells probably exert an immune-modulatory effect within the TME. FRCs cluster closely to the immunomodulating cells and highly express chemokines *CCL2*, *CCL8*, *CXCL2*, *CXCL12* as well as *CCL19* and *CCL21*. The latter two chemokines regulate lymphocyte homing and are characteristic of lymph node FRCs [50–52]. FRCs which are usually present in mucosal, skin and lymph node tissue were accordingly most abundant in tumor-free lymph nodes (on average 18% vs 6% in metastatically affected lymph nodes, Additional file 9: Fig. S9I) [53–55]. Interestingly, we also detected FRCs within the primary tumors, suggesting they are functioning in mucosa-associated lymphoid tissue (MALT, Additional file 9: Fig. S9I, J).

## Discussion

EMT represents the reactivation of an embryonic developmental program in which cells acquire migratory and invasive properties, i.e., prerequisites for invasion and metastasis of cancer [3, 56, 57]. Thus, in early stages of metastasis, tumor cells undergo EMT, whereas in established metastases the reverse process *aka* MET is also observed [58, 59]. To assess the EMP-associated heterogeneity among OSCC cells and gain some insight into the dynamics of this process, we examined the transcriptomes of 7,263 individual carcinoma cells isolated from primary and metastatic OSCC. Although we collected a high number of carcinoma cells in total, one of the limitations of this study is the often low number of malignant cells examined per tumor lesion. Despite this, we were able to demonstrate a progressive MET within a single, established lymph node metastases and confirm the EMP-associated heterogeneity in primary and metastatic OSCC. Interestingly, the epithelial differentiation in OSCC metastases is associated with higher activity of the EMT-activator ZEB1, which was confirmed on protein level by detection of co-expression of ZEB1 and cornifin-B in individual tumor cells using immunofluorescence staining. Consistent with previous reports showing that the EMP status of tumor cells influences the properties of CAFs and vice versa, we also detected distinct CAF phenotypes in primary tumors and tumor-involved lymph nodes; interestingly, immunomodulating fibroblasts were found throughout the metastatic cascade [5, 6].

EMP appears to be the main driver of cellular heterogeneity within OSCC: detailed phenotyping of cancer cells identified several clusters whose predominant functional phenotypes corresponded to different EMP states, ranging from a pEMT to a more epithelial differentiated state. Moreover, pEMT phenotypes in particular might superimpose with traits related to angiogenesis, ECM remodeling, metabolic adaptations, stress, and interactions with the immune system. Metabolic adaptations include response to environmental limitations such as hypoxia and low glucose. Low glucose conditions are counteracted with upregulation of genes related to amino acid metabolism that fuel into glycolysis [40]. While previous studies suggested that the activity of specific metabolic pathways in OSCC varies widely among patients [60], we observed that hypoxia- and stress-related gene expression patterns are similar between patients, supporting the notion of a reactive response rather than an aspect of individual tumor evolution.

In terms of EMP dynamics, it is assumed that cells may transit from one EMP state to another along a continuous spectrum of changes. Currently, however, it is also discussed if long-lived phenotypes representing discrete EMP states prevail [3, 7, 8, 10, 11, 13–15]. Most studies supporting continuous transitions are based on in vitro or preclinical in vivo models that may not fully reflect the complexity of the tumor and its microenvironments [7, 8, 11]. Indeed, human in situ or ex vivo studies suggested distinct EMP states; however, these approaches do not fully capture cellular dynamics [10, 14, 57]. We demonstrated that within each patient, the EMP-driven differences are most prominent and epithelial differentiated phenotypes are profoundly different from pEMT clusters, suggesting that these states may be more static. Moreover, gene expression dynamics estimated by RNA velocity demonstrated epithelial differentiated cells were strongly developing towards a more pronounced epithelial differentiation with an increasing expression of genes of the epidermal differentiation complex [46]. Of note, OSCC cells with a pEMT phenotype did not show such a uniform developmental direction. The assumption that epithelial differentiated metastatic cells developed later than pEMT cells, i.e., underwent MET, is supported by an increasing number of inferred copy number gains towards increasing epithelial differentiation even if accounting for the limitations of this approach.

However, we cannot conclude whether MET happened within the metastasis or primary malignancy, as our data reflects the tumor heterogeneity within a specific timepoint of tumor evolution. As we observed a similar EMP heterogeneity in primary tumors, multiple disseminated tumor cells reflecting this heterogeneity might have migrated collectively, which could be crucial for metastatic consolidation [61].

Unexpectedly, we found high transcriptional activity of the EMT-activator ZEB1 in epithelial differentiated OSCC cells in both primary and metastatic tumor lesions, even considering that the scRNAseq data inferred transcription factor activities are biased towards transcriptional repressors. In the case of primary tumors this may be interpreted as the incipient EMT, but this hypothesis would not work for metastatic lesions where ZEB1 activity was associated with a progressive epithelial differentiation. Indeed, in previous studies, depletion of ZEB1 was reported to drive tumor cells from pEMT towards an epithelial phenotype [62, 63]. However, depletion of Zeb1 in a mouse model also reduces phenotypic variability of cancer cells, particular their phenotypic/metabolic plasticity [62]. While it is well established that ZEB1 together with microRNAs stabilizes EMT through a feedforward loop, this loop could also induce epithelial differentiation based on environmental factors [64]. In addition to the transcriptional repressor activity, ZEB1 has been demonstrated to induce the epithelial differentiation marker cornifin-B in response to IL-1β and IFN-γ [65]. We not only demonstrate the simultaneous occurrence of *SPRR1B* mRNA expression and ZEB1 activity, but also the co-localization of cornifin-B and the ZEB1 protein expression in OSCC lymph node metastases. Thus, although ZEB1 activity is crucial for the induction of the pEMT state, it does not seem to completely prevent partial epithelial differentiation. Remarkably, no relevant differences in *CDH1* expression were detected between the different EMP states in the metastatic OSCC lesions. Therefore, the more epithelial differentiated phenotypes we observed most closely correspond to a partial epithelial differentiation analogous with the observed pEMT phenotypes. We speculate that the driving force behind this EMP-associated heterogeneity of OSCC cells is to maintain cellular integrity. For example, ZEB1 is an ATM-substrate linking ATM and CHK1, promoting homologous recombination-dependent DNA repair and thereby protecting cells from genotoxic stress whereas expression of keratin intermediate filaments helps to protect cells from stress associated apoptosis [66, 67].

Similar to previous reports, we detected various fibroblast phenotypes in OSCC lesions, of which, remarkably, the immunomodulatory *CXCL14*-expressing fibroblasts were found in both primary tumors and lymph node metastases [10, 17, 55, 68]. This indicates the special importance of this subgroup, as they may enable tumor cells to escape from the immune system. Using single-cell mRNAseq data, *CXCL14*-expressing fibroblasts have previously been detected in HNSCC, melanoma, and lung cancer lesions and are presumed to have immunosuppressive effects; the latter explained the association of their presence with poorer prognosis [68]. However, CXCL14 is also constitutively expressed and secreted by fibroblasts and keratinocytes in healthy skin and mucosa [69, 70]. Indeed, the effects of the chemokine CXCL14 seem to depend strongly on the cellular context [71]. For example, restored *CXCL14* expression in HPV-positive oropharyngeal carcinoma is associated with better survival in immunocompetent syngeneic mice [72] but *CXCL14*-producing CAFs promoted tumor growth in a prostate cancer model [73]. Similarly, ECM-modifying and contractile fibroblasts can promote or suppress tumor progression by consolidating or disrupting tissue structure, as ECM remodeling can affect both tumor and immune cell migratory ability [74].

Our study demonstrates that the comprehensive molecular characterization of tumor lesions captures both their complexity, as well as the heterogeneity between manifestations and the dynamics of their cellular composition [75]. In particular, the heterogeneity of EMP status in HNSCC appears to be of translational importance as it provides further insight into tumor aggressiveness and treatment resistance. Similarly, it may help to assess the impact of systemic therapies on the microenvironment and correlate different EMP phenotypes with further clinical progression [76]. This also applies to the effect of perioperative drugs, which are designed to counteract the spread of cancer by inhibiting stress-inflammatory responses such as the release of catecholamines and prostaglandins [77]. Patients would therefore benefit from translational molecular companion programs by differentiating early effective from ineffective interventions.

In summary, the data presented here indicates that the interplay between tumor and stromal cell interactions is a highly complex process and that the EMP status of tumor cells and the polarization of stromal cells may influence each other. Our observations suggest that tumor cells and CAFs behave similarly in primary and metastatic OSCC samples. These findings may help to unravel the role of fibroblasts in predicting metastasis risk, which in turn may influence treatment decisions in OSCC.

## Conclusions

Single cell transcriptomics reveals that heterogeneity within OSCC cells is dominated by EMP differences resulting in distinct partial EMT and epithelial differentiated phenotypes. Particularly, the partial EMT phenotypes can be accompanied by features related to metabolic adaptations, stress, and interaction with the immune system. In addition, CAFs were shown to be a major component of the TME, with immunomodulating *CXCL14*-expressing fibroblasts in both primary OSCC tumors and lymph node metastases indicating their relevance during immune escape. The EMP phenotypes likely endow capabilities that are essential for the different stages of the metastatic process, including maintenance, cellular integrity and polarization of stromal cells. This could be a possible additional function of ZEB1, as it is also expressed during progressive epithelial differentiation in OSCC metastases.

### Supplementary Information

**Additional file 1: Figure S1.** Histology of OSCC primary and metastatic tumors. Whole-slide image H&E staining for FFPE sections of OSCC samples. Scale bars depict 2 mm. High-resolution pictures are available through DOI: https://doi.org/10.6084/m9.figshare.20905837.v1.

**Additional file 2: Figure S2.** Cell type identification by marker genes, automated reference-based annotation, differential expression and inferred CNVs. (**A**) Expression of marker genes (x-axis) for each cell type (y-axis) in our cohort. Dots are colored by the average log-normalized gene expression and the dot size represents the percentage of cells with detected expression of the respective gene within the cell type. (**B**) UMAP of 41,284 cells from our cohort cells colored by SingleR annotations using the Monaco bulk RNA dataset on shared-nearest neighbor clusters with resolution 100. (**C**) Heatmap for scaled, lognormalized gene expression of all cell types from patient #1 and their top 10 DEGs (rows) against all other cells. DEGs are sorted from highest to lowest log2 foldchange. (**D**) Inferred CNVs across cells (rows) of different cell types without mitochondrial genes from patient #1. Columns show genes categorized in chromosomes and ordered by genome position; hence the size of the chromosome reflects the number of detected genes and not its nucleotide length. (**E**) Standard deviation of the log2 inferCNV values to the mean of non-malignant cells compared between non-malignant and malignant cells of patient #1.

**Additional file 3: Figure S3.** PEMT and epithelial differentiating gene expression signatures are comparable to previously published EMT signatures. (**A**) EMT hallmark gene set enrichment plot for log2 fold changes of pEMT cells against all other cells of lymph node metastasis from patient 1. Shown is the stepwise calculated enrichment score, black lines indicate genes present in the respective gene set. (**B**) Average log2 fold change of gene expression (x-axis) and differences in cellular fractions expressing the respective gene (y-axis) between pEMT and epithelial differentiated cell clusters. Labelled in red are genes with log2 foldchange below or above 1 that are included in the epithelial differentiation or pEMT signature, respectively, with top 10 genes named. The histogram on top shows the number of genes across the log2 fold change with in total 100 bins. (**C**, **D**) Average expression scores (y-axis) of the pEMT (**C**) and epithelial differentiation (**D**) signatures across tumor phenotypes from patient #1 depicted in figure 2A (xaxis) color-coded by these clusters. (**E**) Heatmap of correlation coefficients of GSVA scores between 91 EMP-related signatures of malignant cells, derived from the EMTome database and selected publications (9, 10, 14). On the right side, the correlation coefficients between GSVA scores of EMT signatures from the EMTome database and of epithelial differentiation and pEMT signatures from patient #1 (right) are shown and next to it, annotated as ”EXPR_perc”, is the fraction of genes with non-zero expression and the size of the respective EMT signature in log10 scale with the respective number next to it. Rows and columns are hierarchically clustered using a spearman correlation distance (1-cor(x,y)) and ward.D2 method.

**Additional file 4: Figure S4.** Extended analysis of the tumor phenotype characterization for the lymph node metastasis of patient #1. (**A**) Top 5 enriched gene sets from log2 foldchanges of respective tumor phenotypes by normalized enrichment scores (x-axis). Gene sets of respective phenotypes are sorted from highest to lowest enrichment. Bars are colored by the negative decadic logarithm of the Benjamini- Hochberg adjusted p-value (padj). (**B**) First two PCs of OSCC cells with all six principal curves that are derived from trajectory inference. Graph on top visualizes the relationship between malignant phenotypes, described by the principal curves forming a branching trajectory. Cells responding to environmental conditions form their own branch, indicating that the strong reactive response determines their predominant phenotype. (**C**) Inferred CNVs across tumor cells of patient #1 (rows) for all chromosomes (columns). Columns show genes categorized in chromosomes and ordered by genome position; hence the size of the chromosome reflects the number of detected genes and not its nucleotide length. Mitochondrial genes were excluded. (**D**, **E**) Inferred CNVs across tumor cells (rows) of chromosome 1 (**D**) and chromosome 17 (**E**) showing genes (columns) ordered by genome position. The signal on chromosome 1 is located on a genomic position on which S100 genes are accumulating and the signal on chromosome 17 on a location with accumulation of cytokeratins; most of these genes are highly expressed in the more epithelial differentiated cells.

**Additional file 5: Figure S5.** Malignant phenotypes characterized across all analyzed patients. (**A**) Number of cells (y-axis) for each library (x-axis) showing the cells that are used for 10x Genomics scRNAseq (light blue) and all recovered, i.e., detected, cells after sequencing (blue). Based on manufacturers information a recovery rate around 50% is expected. (**B**) Heatmap for scaled, log-normalized gene expression of tumor cells (columns) split by respective phenotype depicted in Figure 3C and the top 10 DEGs (rows) of the respective phenotype against all other tumor cells. DEGs are sorted from highest to lowest log2 foldchange and row sections are ordered the same as column section. On bottom, the respective patient and localization is annotated for each cell. (**C**) Top 5 enriched gene sets from log2 foldchanges of respective tumor phenotypes by normalized enrichment scores (x-axis). Gene sets of respective phenotypes are sorted from highest to lowest enrichment. Bars are colored by the negative decadic logarithm of the Benjamini- Hochberg adjusted p-value (padj). (**D**) UMAP of OSCC cells as depicted in figure 3C with PCs corrected for patient-specific effects using harmony. Cells are annotated according to their patient id. (**E**) Inferred CNVs across EMP-related OSCC cells from patient HN01 (rows) for all chromosomes (columns). Cells split by their EMP phenotype do not show any differences in their inferred CNVs pattern. Columns show genes categorized in chromosomes and ordered by genome position; hence the size of the chromosome reflects the number of detected genes and not its nucleotide length. Mitochondrial genes were excluded. (**F**) UMAPs of malignant cells from all respective patients. Cells are annotated SNN clusters and renamed according to the predominant phenotype. (**G**) Same plot as depicted in Figure 3F with the names of all patient-specific clusters as shown in E followed be the patient id.

**Additional file 6: Figure S6.** Inferred transcription factor activity might be biased by activator or repressor function. (**A**) Distribution of the mean activity of all cells from patient #1 for all transcription factors split by repressor, ambiguous and activators. Repressors and activators are defined based on more than 90% of the target genes being either repressed or upregulated, transcription factors with less than 90% for both are in the ambiguous class. (**B**) Distribution of the fraction of cells within a respective cell cluster with a transcription factor activity of greater than 0. The clusters include all cell types and malignant cell clusters from patient #1 split by activators, ambiguous and repressors. Clusters with high fraction of cells with activity greater than 0 indicate an active transcription factor, which is more prominent across repressors than for activators.

**Additional file 7: Figure S7.** Cell type abundances across patients and tissues. (**A**) Relative fractions (xaxis) of cell types (y-axis) across different patients (left) or tissue types (middle) with the absolute number of cells per cell type (right), colored by cell types from Figure 5C. “NA” denotes cells that could not be demultiplexed from hashed samples and hence could not be assigned to a tissue type. (**B**) Relative fractions (x-axis) of cell types (y-axis) across different patients (left) with absolute numbers per cell type (right). (**C**) UMAP of 41,284 cells based on OSCC scRNAseq data from our cohort and colored by patients. (**D**) UMAP of 21,037 cells based on CD45-negative and HPV-negative primary HNSCC from Kürten et al. and colored by patients.

**Additional file 8: Figure S8.** Bulk transcriptomes reveal the cellular composition of OSCC across tissue types. (**A**) Fractions of cell types (x-axis) across all samples (y-axis) including primary tumors (PT), metastatic lymph nodes (MET) and tumor-free lymph nodes (LN) for cells detected by scRNAseq (left panel) or deconvoluted from bulk transcriptome analysis (right panel). (**B**) Pie charts showing the average fraction of celltypes across samples from each tissue type, derived from scRNAseq data (top) and bulk transcriptome deconvolution (bottom) and colored by cell type. cDCs: conventional dendritic cells; pDCs: plasmacytoid dendritic cells; RBCs: red blood cells; ECs: endothelial cells.

**Additional file 9: Figure S9.** Characterization of OSCC-derived fibroblasts. (**A**) Heatmap of scaled, log-normalized expression of the top 5 differentially expressed genes (DEGs) (rows) for fibroblasts and pericytes (columns) split by their respective phenotype. DEGs are sorted from highest towards lowest log2 foldchange and row sections are ordered like column sections. (**B**) Normalized enrichment scores (NES) of top 5 enriched gene sets for each fibroblast phenotype. Gene sets are sorted from highest to lowest NES and the bar chart is colored by negative decadic logarithm of Benjamini-Hochberg adjusted p-values (padj). (**C**) Scaled, log-normalized expression of collagens (COL) (rows) across fibroblasts and pericytes split by respective phenotypes (columns). Rows are clustered by their similarity using the Euclidean distance and ward.D2 method. (**D**) Selected genes (y-axis) expressed across phenotypes (x-axis). Dots are colored by averaged log-normalized gene expression and dot size represents the percentage of cells expressed in this phenotype, i.e., cells with more than 1 unique molecular identifier (UMI) detected in the respective gene. (**E**-**H**) Analog to AD for fibroblasts and pericytes from Kürten et al. dataset. (**I**) Composition of phenotypes across tissue types in pie charts (top) and across samples as bar chart (bottom). Pie charts show the average fraction of phenotypes across fibroblasts and pericytes for each tissue type. The bar chart shows the fraction of phenotypes (x-axis) across samples (y-axis) on the left with the absolute abundance of cells on the right side, colored by tissue type. (**J**) Similar plot as in I for the Kürten et al. dataset. As all samples represent primary tumors, they were summarized in one pie chart.

**Additional file 10: Table S1.** Clinical and sequencing information from OSCC patients.

**Additional file 11. **Materials and Methods.

## Data Availability

The processed datasets generated and analyzed during the current study are available in the GEO database with accession id GSE195655. The raw FASTQ files are available upon request due to privacy reasons. The high-resolution H&E Images are available through figshare under the https://doi.org/10.6084/m9.figshare.20905837.v1. The publicly available FASTQ files from Kürten et al*.* were downloaded from the sequencing read archive (SRA) accession id SRP301444. Code used for analysis is available at https://github.com/sci-kai/single_cell_EMP.

## Abbreviations

CAF: Cancer-associated fibroblast
CNV: Copy number variation
DC: Dendritic cell
DEG: Differentially expressed genes
EC: Endothelial cell
ECM: Extracellular matrix
EMP: Epithelial–mesenchymal plasticity
EMT: Epithelial–mesenchymal transition
FFPE: Formalin-fixed, paraffin-embedded
GSEA: Gene set enrichment analysis
GSVA: Gene set variation analysis
HNSCC: Head and neck squamous cell carcinomas
HPV: Human papillomavirus
HTO: Hashtag oligo
IHC: Immunohistochemistry
MET: Mesenchymal-epithelial transition
MSigDB: Molecular signature database
OSCC: Oral cavity squamous cell carcinoma
PC: Principal component
pEMT: Partial EMT
scRNAseq: Single-cell RNA sequencing
SNN: Shared-nearest neighbor
TME: Tumor microenvironment
UMAP: Uniform manifold approximation and projection
UMI: Unique molecular identifier
