# Supplementary figures and images for: Mesenchymal–epithelial transition in lymph node metastases of oral squamous cell carcinoma is accompanied by ZEB1 expression
Source: J Transl Med. 2023 Apr 19;21:267. doi: 10.1186/s12967-023-04102-w (PMC10114373; doi:10.1186/s12967-023-04102-w)

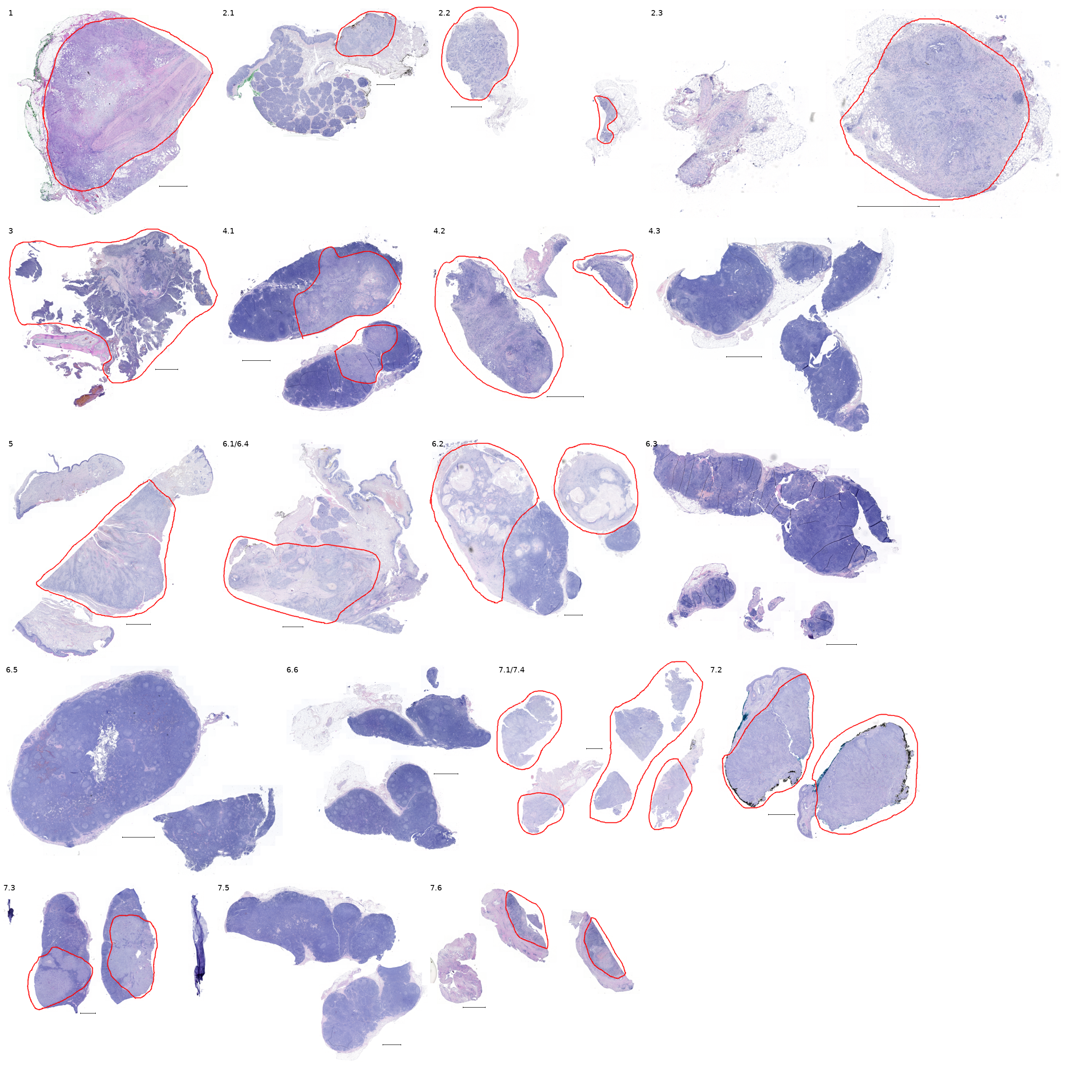

Supplement: Supplementary file 1 — Additional file 1: Figure S1. Histology of OSCC primary and metastatic tumors. Whole-slide image H&E staining for FFPE sections of OSCC samples. Scale bars depict 2 mm. High-resolution pictures are available through DOI: https://doi.org/10.6084/m9.figshare.20905837.v1. [file 12967_2023_4102_MOESM1_ESM.png]

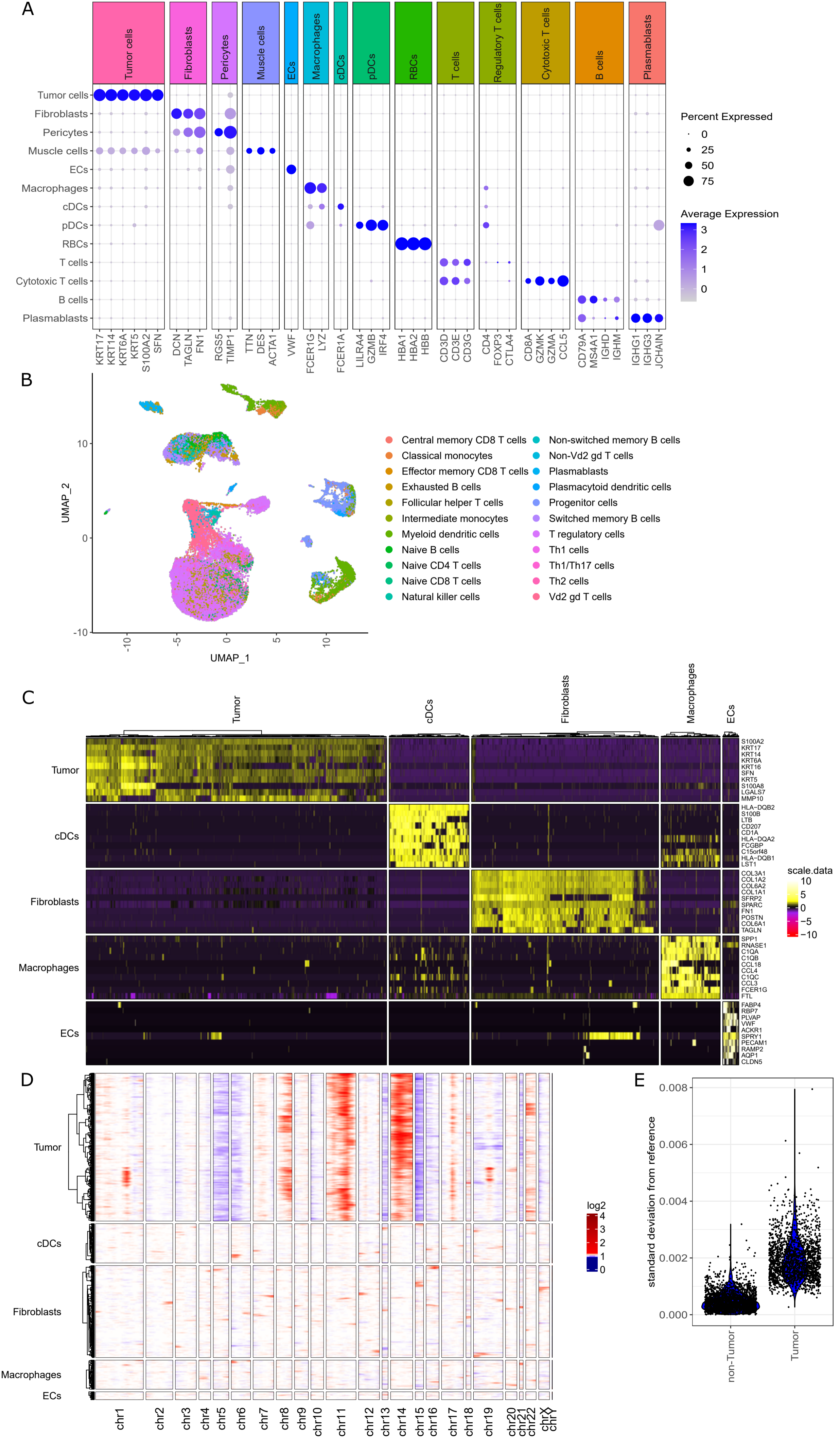

Supplement: Supplementary file 2 — Additional file 2: Figure S2. Cell type identification by marker genes, automated reference-based annotation, differential expression and inferred CNVs. (A) Expression of marker genes (x-axis) for each cell type (y-axis) in our cohort. Dots are colored by the average log-normalized gene expression and the dot size represents the percentage of cells with detected expression of the respective gene within the cell type. (B) UMAP of 41,284 cells from our cohort cells colored by SingleR annotations using the Monaco bulk RNA dataset on shared-nearest neighbor clusters with resolution 100. (C) Heatmap for scaled, lognormalized gene expression of all cell types from patient #1 and their top 10 DEGs (rows) against all other cells. DEGs are sorted from highest to lowest log2 foldchange. (D) Inferred CNVs across cells (rows) of different cell types without mitochondrial genes from patient #1. Columns show genes categorized in chromosomes and ordered by genome position; hence the size of the chromosome reflects the number of detected genes and not its nucleotide length. (E) Standard deviation of the log2 inferCNV values to the mean of non-malignant cells compared between non-malignant and malignant cells of patient #1. [file 12967_2023_4102_MOESM2_ESM.pdf]

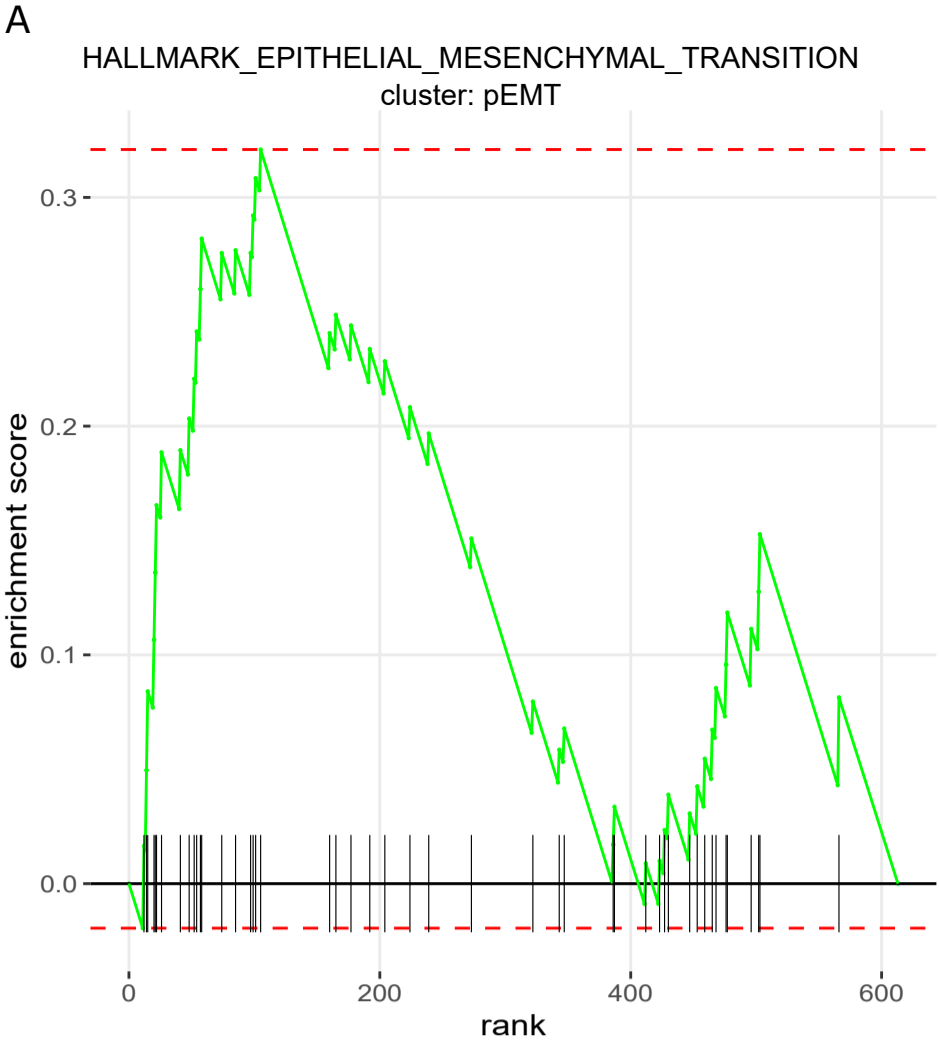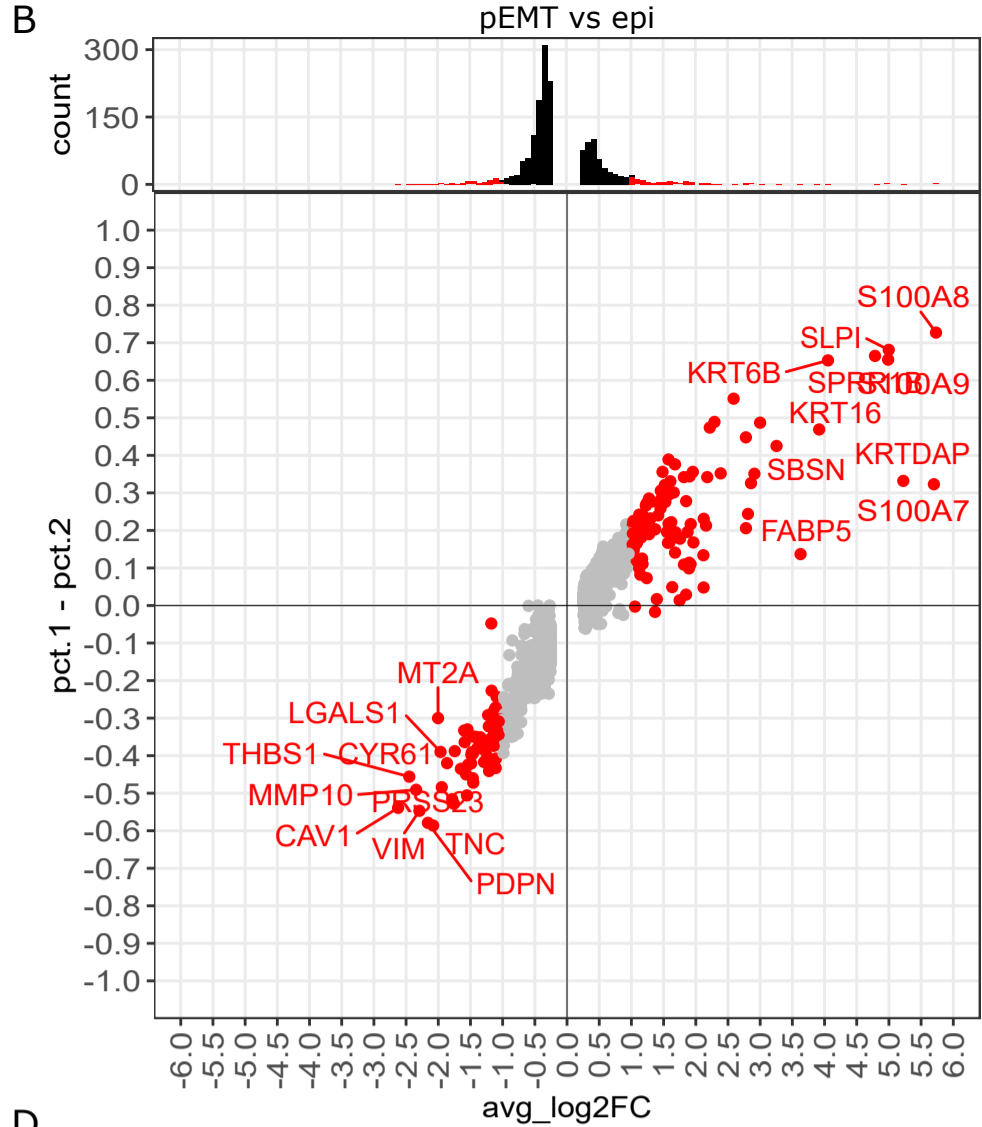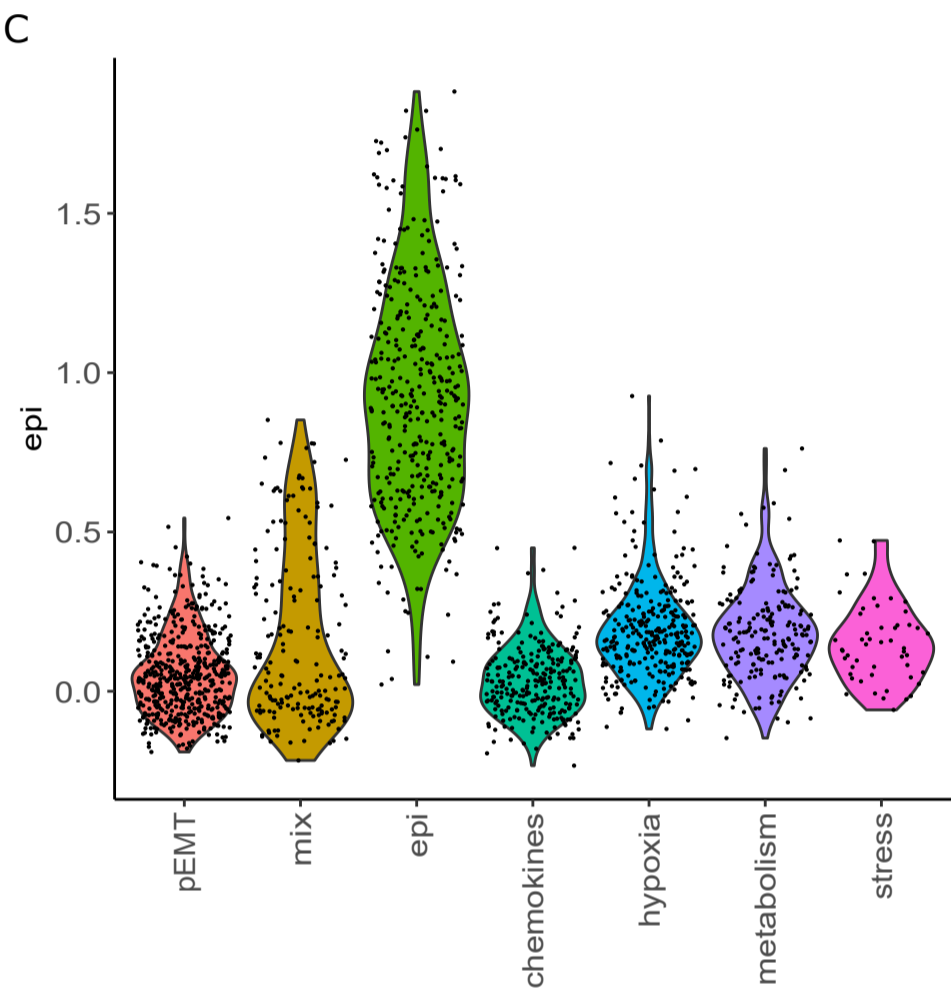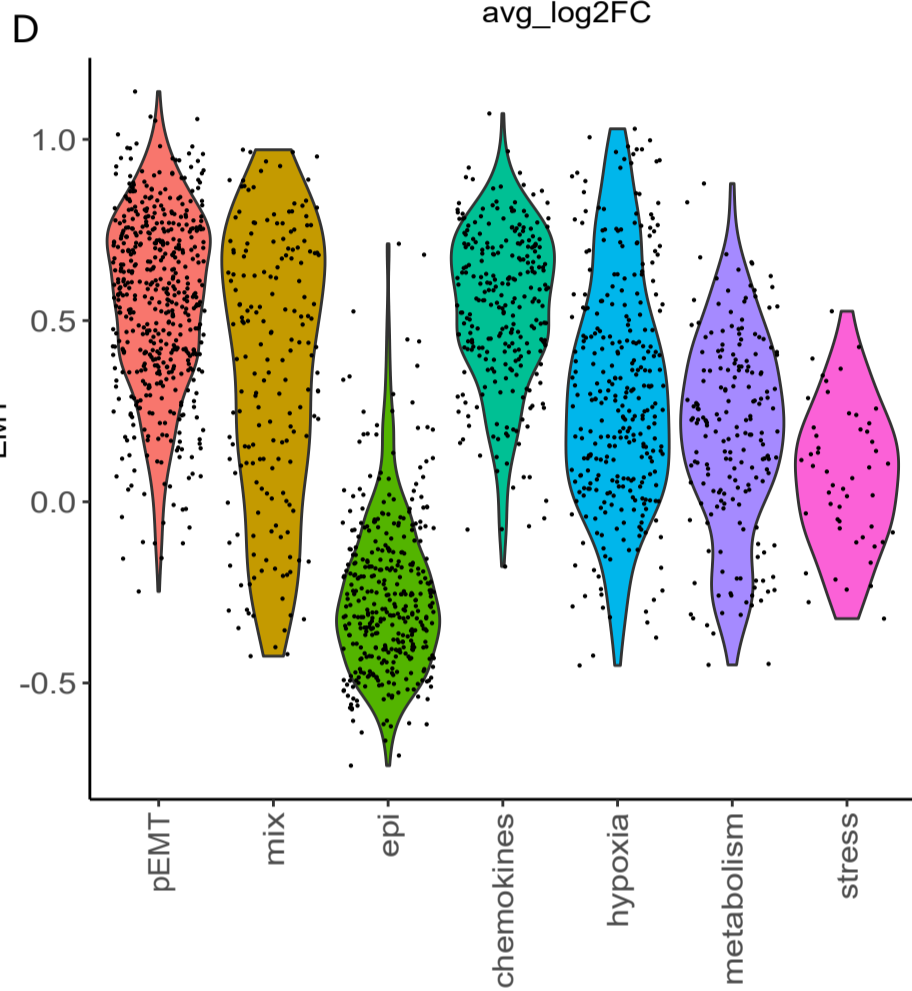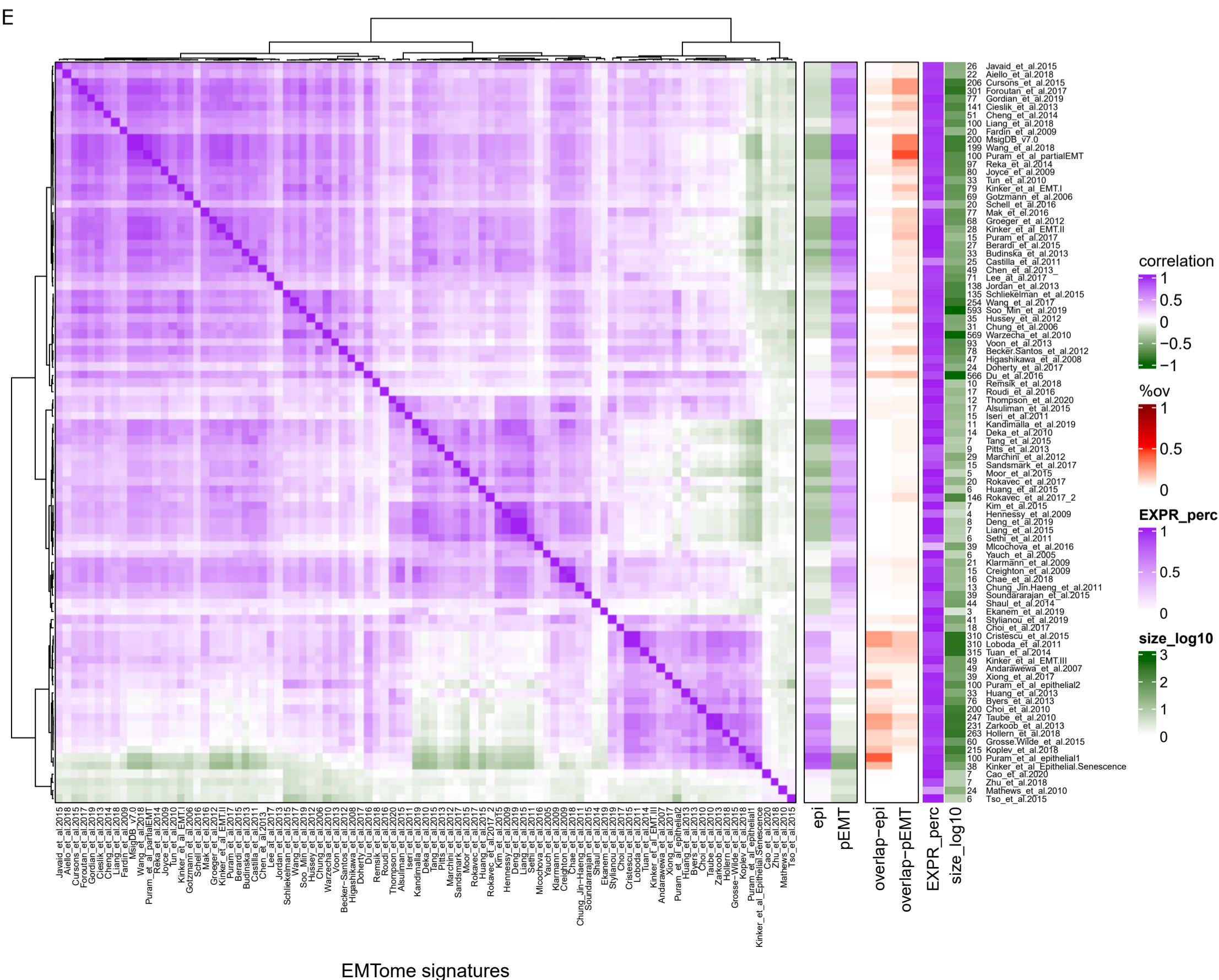

Supplement: Supplementary file 3 — Additional file 3: Figure S3. PEMT and epithelial differentiating gene expression signatures are comparable to previously published EMT signatures. (A) EMT hallmark gene set enrichment plot for log2 fold changes of pEMT cells against all other cells of lymph node metastasis from patient 1. Shown is the stepwise calculated enrichment score, black lines indicate genes present in the respective gene set. (B) Average log2 fold change of gene expression (x-axis) and differences in cellular fractions expressing the respective gene (y-axis) between pEMT and epithelial differentiated cell clusters. Labelled in red are genes with log2 foldchange below or above 1 that are included in the epithelial differentiation or pEMT signature, respectively, with top 10 genes named. The histogram on top shows the number of genes across the log2 fold change with in total 100 bins. (C, D) Average expression scores (y-axis) of the pEMT (C) and epithelial differentiation (D) signatures across tumor phenotypes from patient #1 depicted in figure 2A (xaxis) color-coded by these clusters. (E) Heatmap of correlation coefficients of GSVA scores between 91 EMP-related signatures of malignant cells, derived from the EMTome database and selected publications (9, 10, 14). On the right side, the correlation coefficients between GSVA scores of EMT signatures from the EMTome database and of epithelial differentiation and pEMT signatures from patient #1 (right) are shown and next to it, annotated as ”EXPR_perc”, is the fraction of genes with non-zero expression and the size of the respective EMT signature in log10 scale with the respective number next to it. Rows and columns are hierarchically clustered using a spearman correlation distance (1-cor(x,y)) and ward.D2 method. [file 12967_2023_4102_MOESM3_ESM.pdf]

A

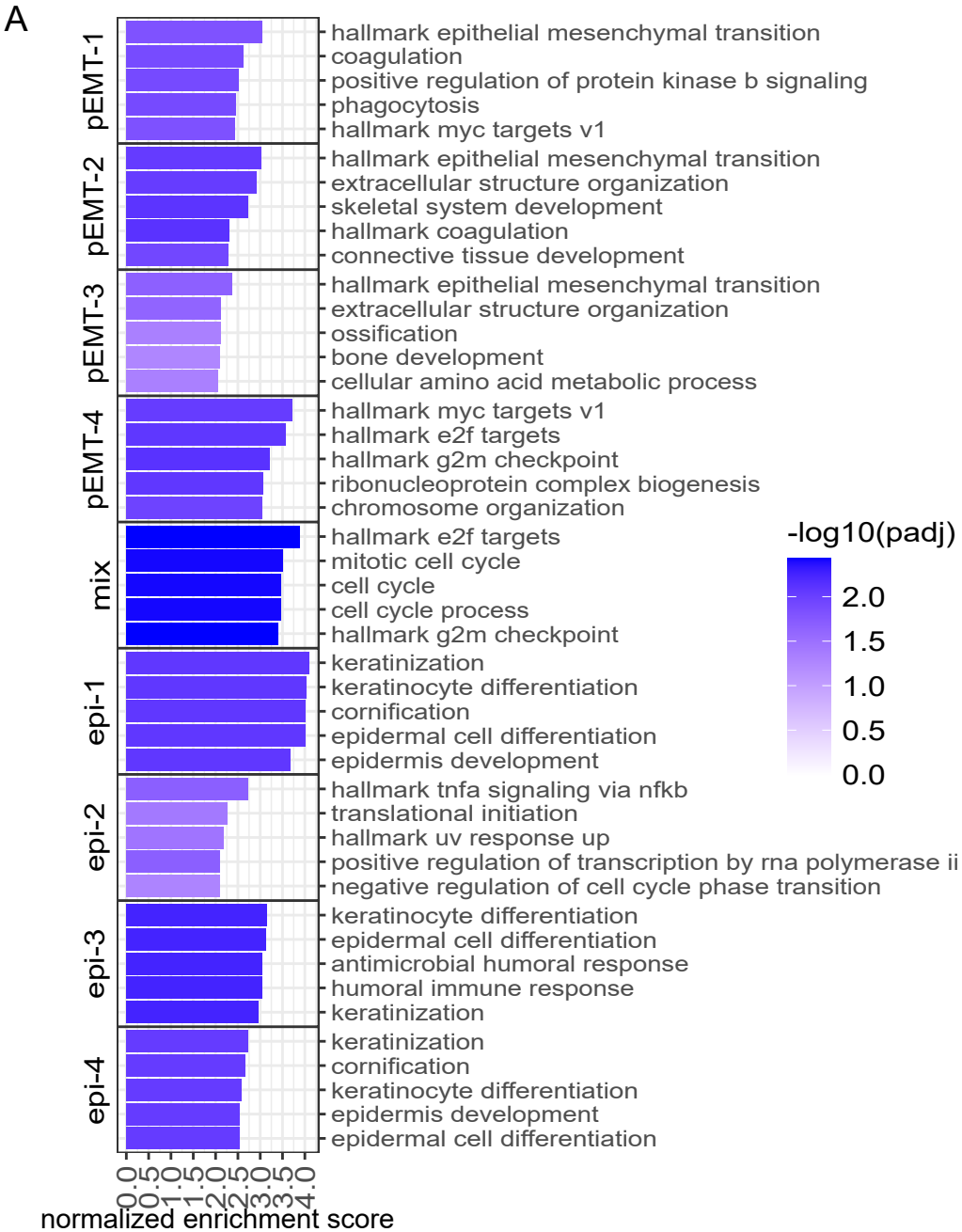

B

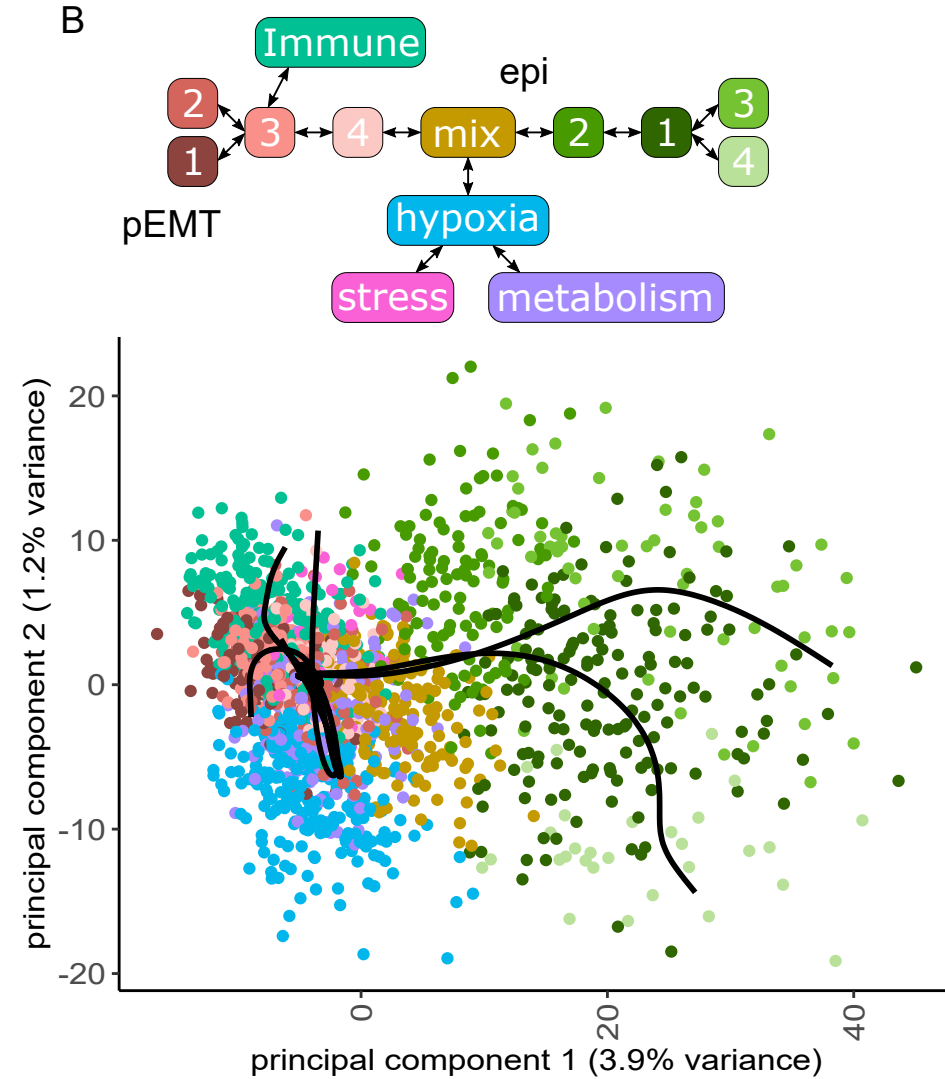

C

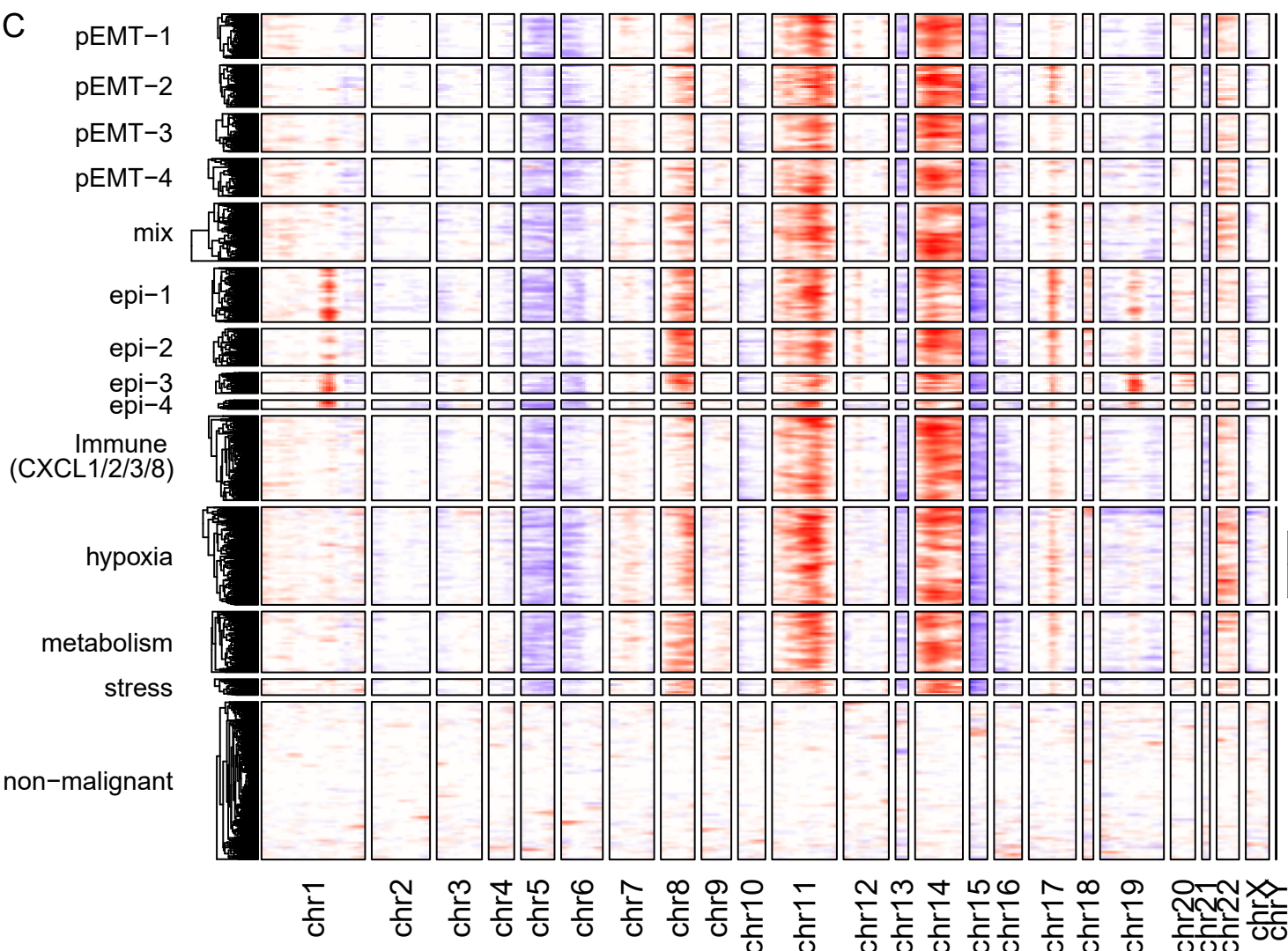

D

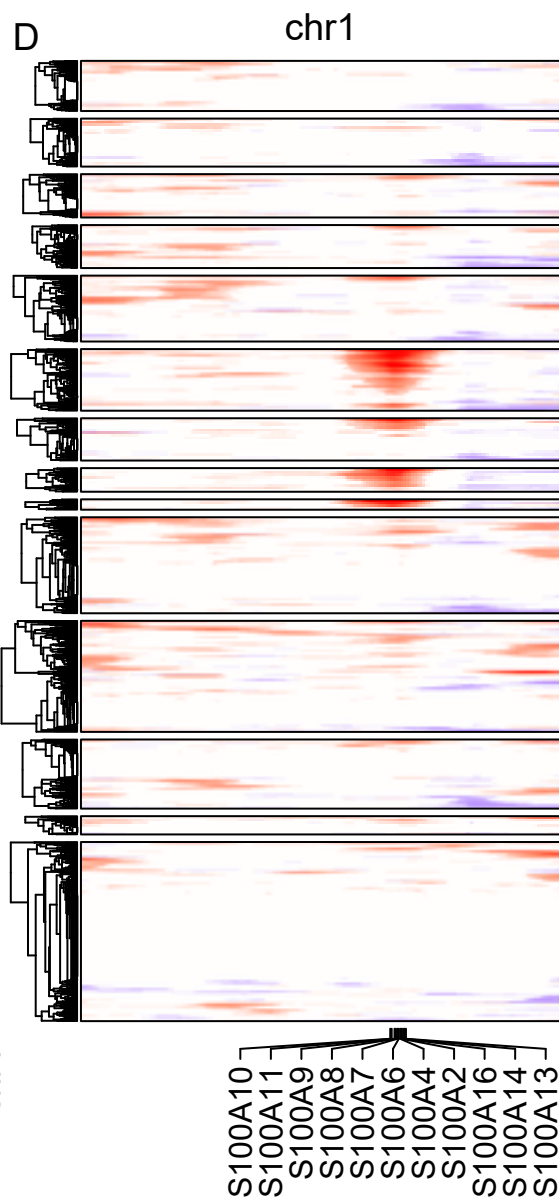

E

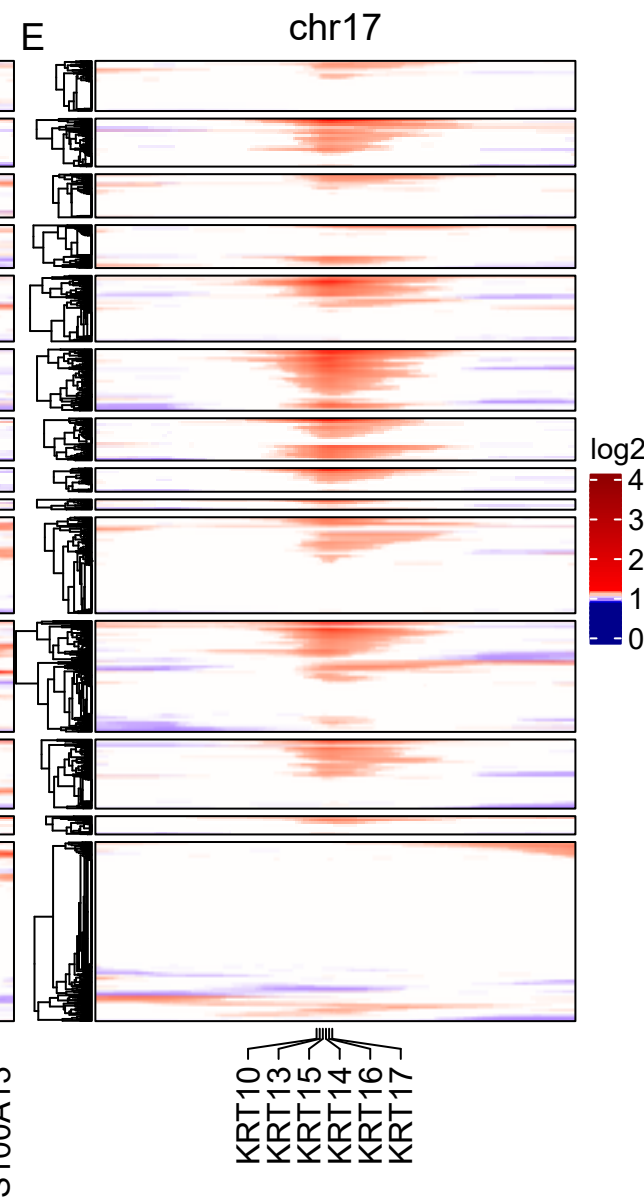

Supplement: Supplementary file 4 — Additional file 4: Figure S4. Extended analysis of the tumor phenotype characterization for the lymph node metastasis of patient #1. (A) Top 5 enriched gene sets from log2 foldchanges of respective tumor phenotypes by normalized enrichment scores (x-axis). Gene sets of respective phenotypes are sorted from highest to lowest enrichment. Bars are colored by the negative decadic logarithm of the Benjamini- Hochberg adjusted p-value (padj). (B) First two PCs of OSCC cells with all six principal curves that are derived from trajectory inference. Graph on top visualizes the relationship between malignant phenotypes, described by the principal curves forming a branching trajectory. Cells responding to environmental conditions form their own branch, indicating that the strong reactive response determines their predominant phenotype. (C) Inferred CNVs across tumor cells of patient #1 (rows) for all chromosomes (columns). Columns show genes categorized in chromosomes and ordered by genome position; hence the size of the chromosome reflects the number of detected genes and not its nucleotide length. Mitochondrial genes were excluded. (D, E) Inferred CNVs across tumor cells (rows) of chromosome 1 (D) and chromosome 17 (E) showing genes (columns) ordered by genome position. The signal on chromosome 1 is located on a genomic position on which S100 genes are accumulating and the signal on chromosome 17 on a location with accumulation of cytokeratins; most of these genes are highly expressed in the more epithelial differentiated cells. [file 12967_2023_4102_MOESM4_ESM.pdf]

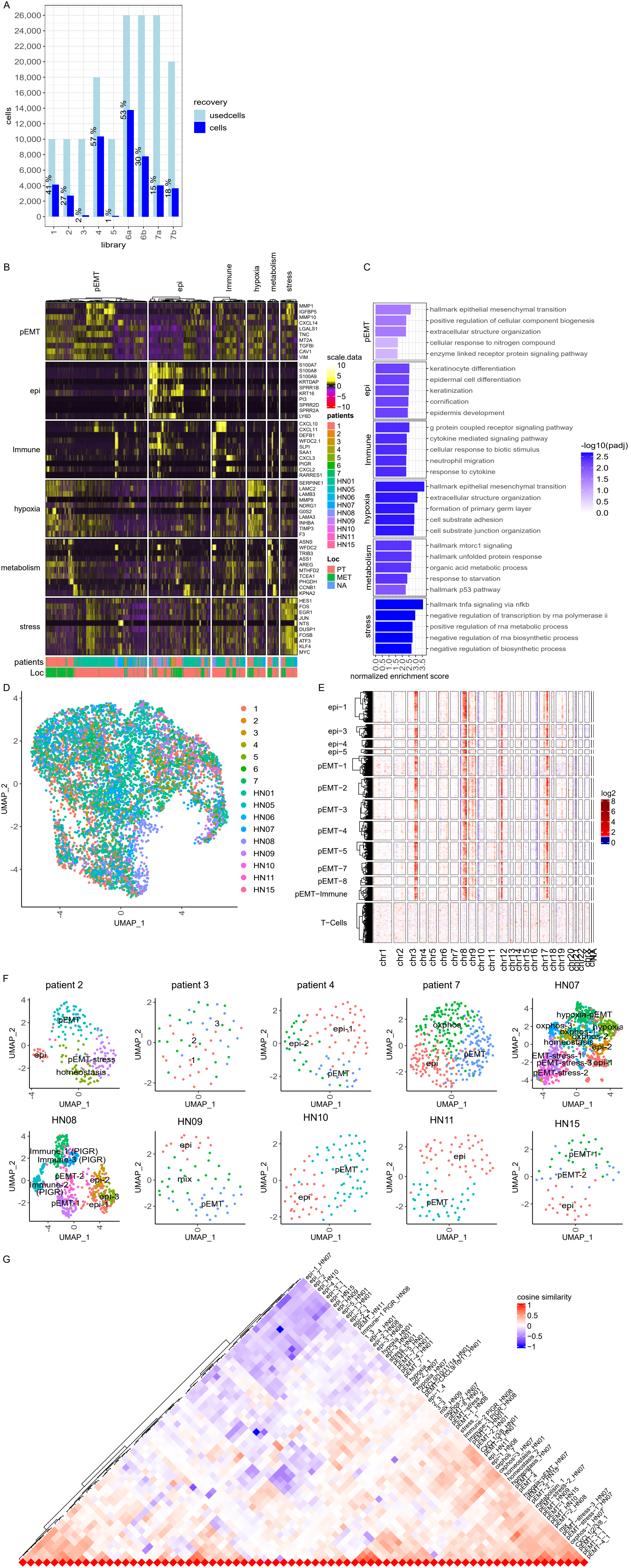

Supplement: Supplementary file 5 — Additional file 5: Figure S5. Malignant phenotypes characterized across all analyzed patients. (A) Number of cells (y-axis) for each library (x-axis) showing the cells that are used for 10x Genomics scRNAseq (light blue) and all recovered, i.e., detected, cells after sequencing (blue). Based on manufacturers information a recovery rate around 50% is expected. (B) Heatmap for scaled, log-normalized gene expression of tumor cells (columns) split by respective phenotype depicted in Figure 3C and the top 10 DEGs (rows) of the respective phenotype against all other tumor cells. DEGs are sorted from highest to lowest log2 foldchange and row sections are ordered the same as column section. On bottom, the respective patient and localization is annotated for each cell. (C) Top 5 enriched gene sets from log2 foldchanges of respective tumor phenotypes by normalized enrichment scores (x-axis). Gene sets of respective phenotypes are sorted from highest to lowest enrichment. Bars are colored by the negative decadic logarithm of the Benjamini- Hochberg adjusted p-value (padj). (D) UMAP of OSCC cells as depicted in figure 3C with PCs corrected for patient-specific effects using harmony. Cells are annotated according to their patient id. (E) Inferred CNVs across EMP-related OSCC cells from patient HN01 (rows) for all chromosomes (columns). Cells split by their EMP phenotype do not show any differences in their inferred CNVs pattern. Columns show genes categorized in chromosomes and ordered by genome position; hence the size of the chromosome reflects the number of detected genes and not its nucleotide length. Mitochondrial genes were excluded. (F) UMAPs of malignant cells from all respective patients. Cells are annotated SNN clusters and renamed according to the predominant phenotype. (G) Same plot as depicted in Figure 3F with the names of all patient-specific clusters as shown in E followed be the patient id. [file 12967_2023_4102_MOESM5_ESM.pdf]

**A**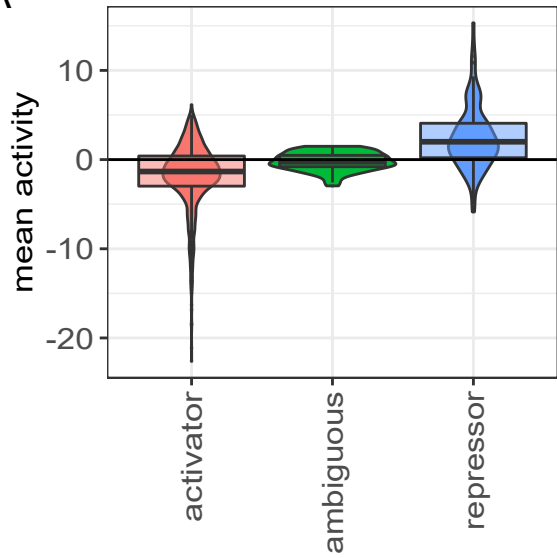**B**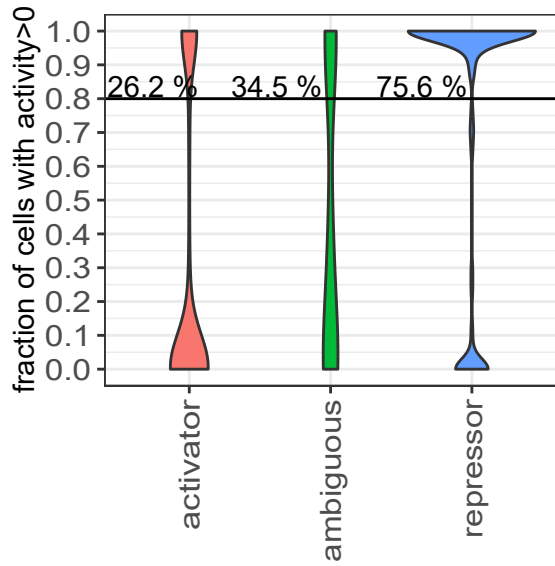

Supplement: Supplementary file 6 — Additional file 6: Figure S6. Inferred transcription factor activity might be biased by activator or repressor function. (A) Distribution of the mean activity of all cells from patient #1 for all transcription factors split by repressor, ambiguous and activators. Repressors and activators are defined based on more than 90% of the target genes being either repressed or upregulated, transcription factors with less than 90% for both are in the ambiguous class. (B) Distribution of the fraction of cells within a respective cell cluster with a transcription factor activity of greater than 0. The clusters include all cell types and malignant cell clusters from patient #1 split by activators, ambiguous and repressors. Clusters with high fraction of cells with activity greater than 0 indicate an active transcription factor, which is more prominent across repressors than for activators. [file 12967_2023_4102_MOESM6_ESM.pdf]

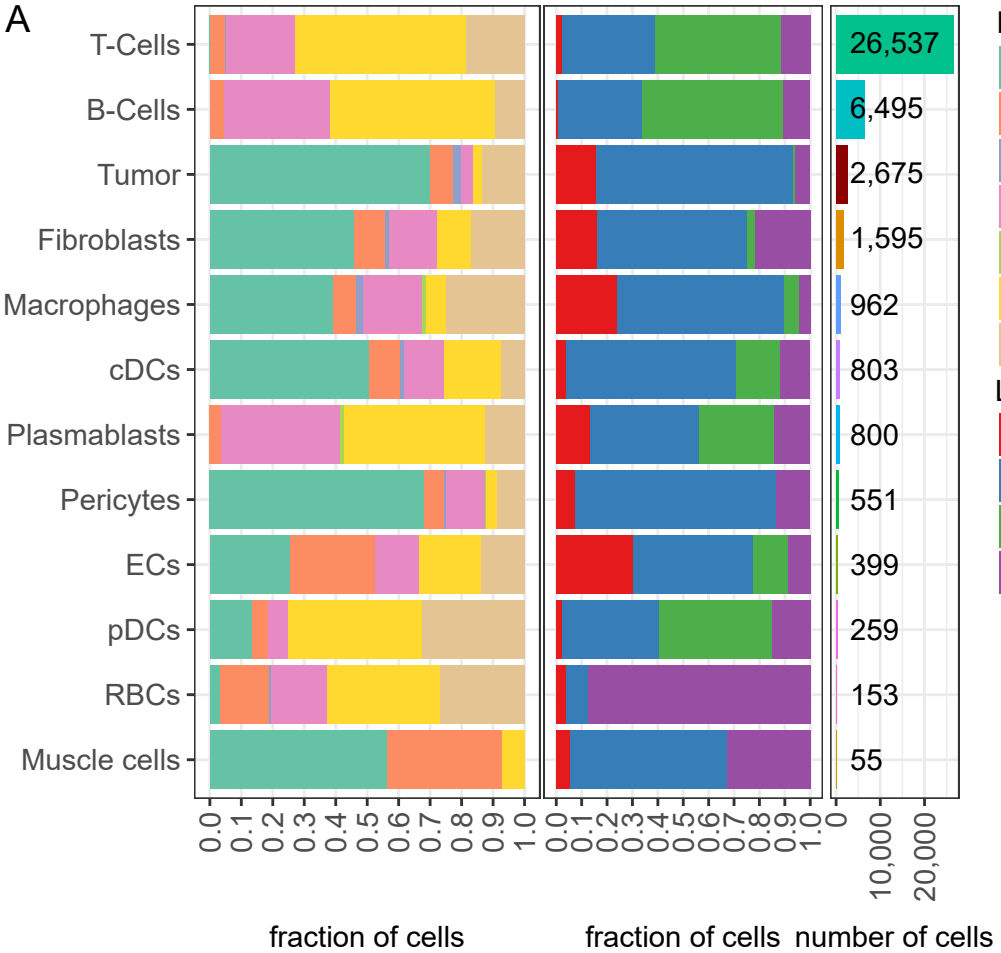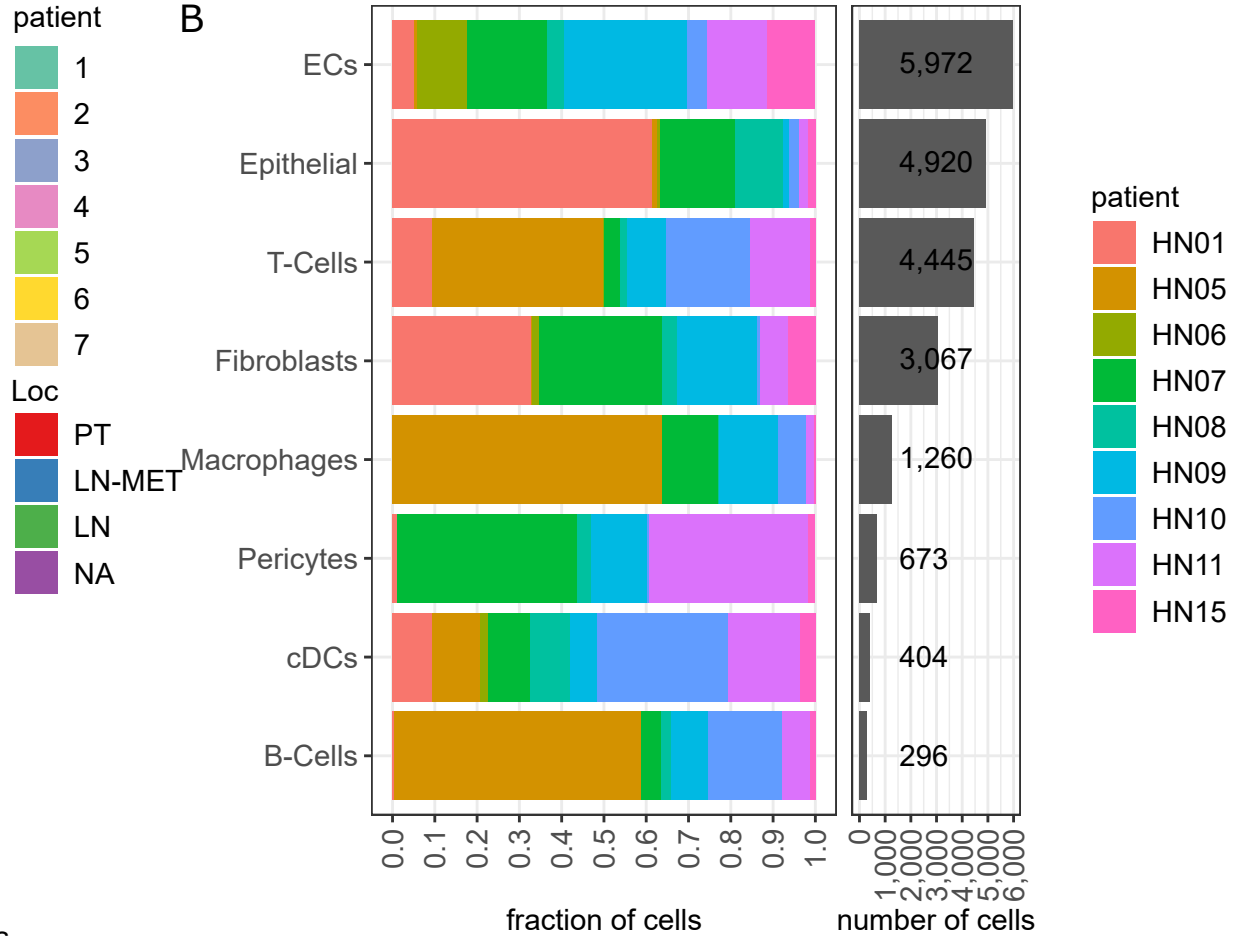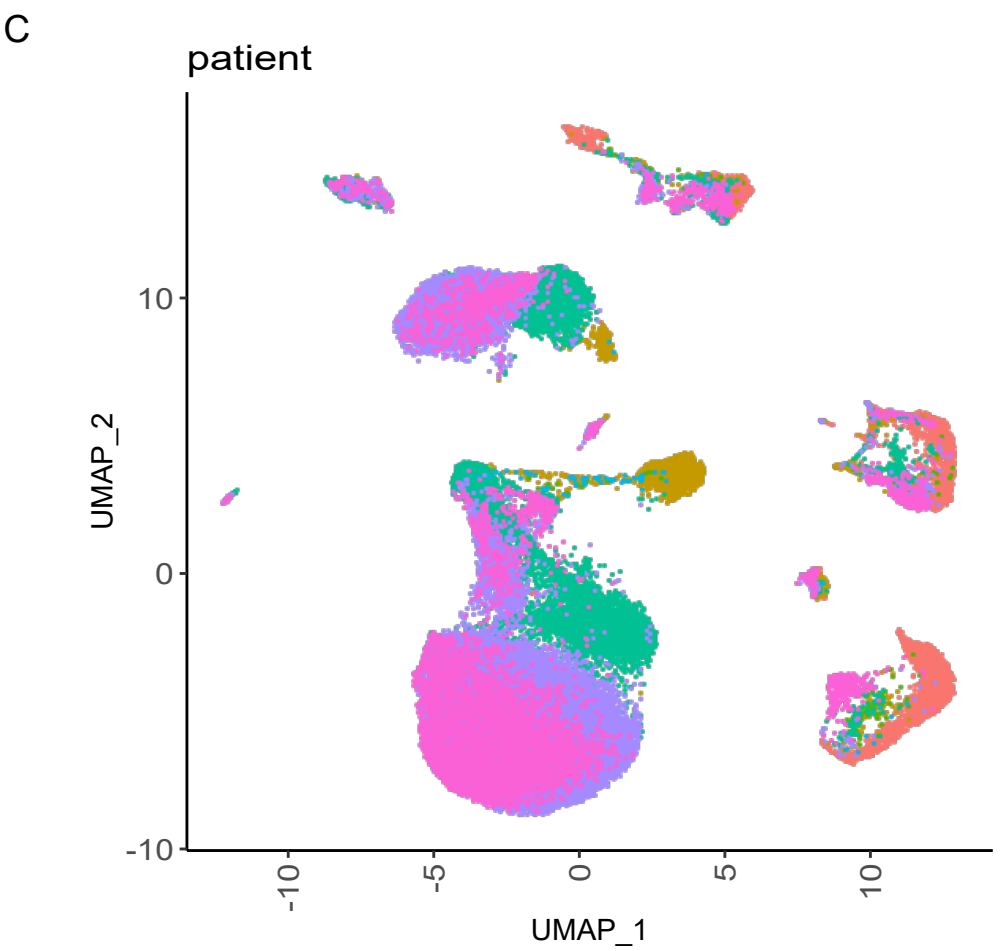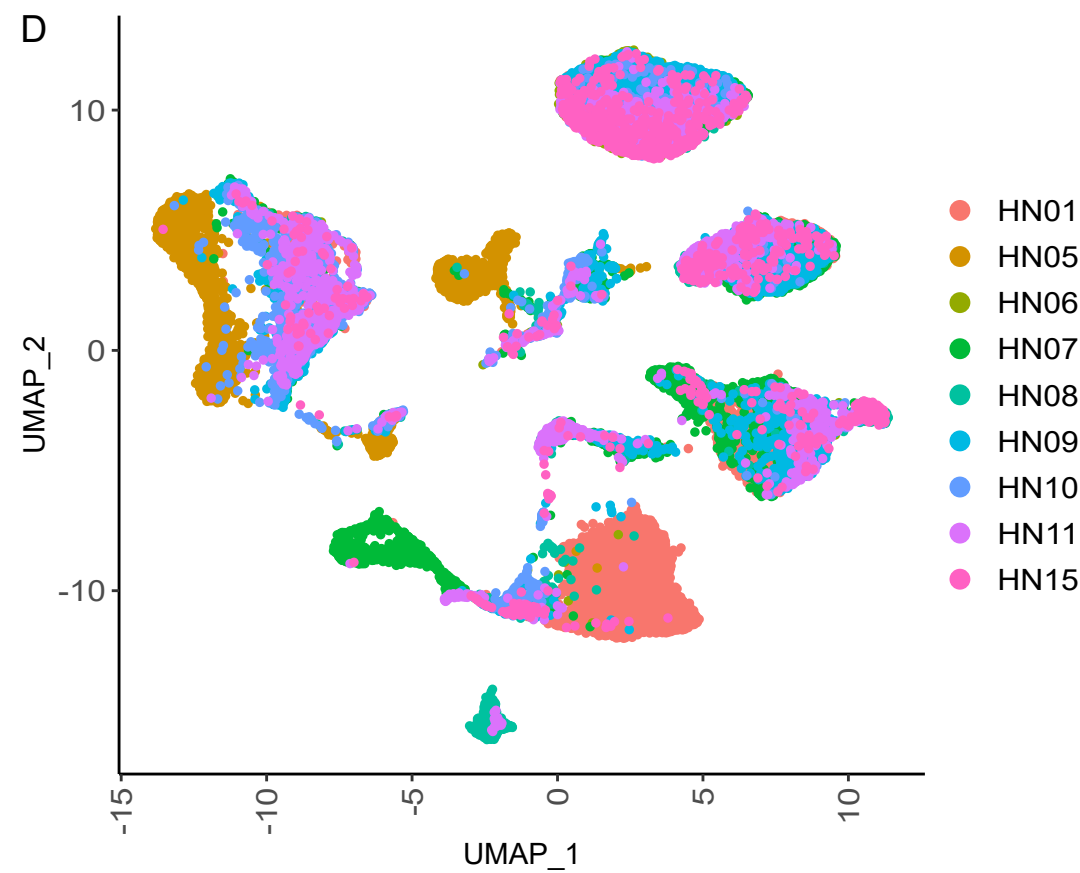

Supplement: Supplementary file 7 — Additional file 7: Figure S7. Cell type abundances across patients and tissues. (A) Relative fractions (xaxis) of cell types (y-axis) across different patients (left) or tissue types (middle) with the absolute number of cells per cell type (right), colored by cell types from Figure 5C. “NA” denotes cells that could not be demultiplexed from hashed samples and hence could not be assigned to a tissue type. (B) Relative fractions (x-axis) of cell types (y-axis) across different patients (left) with absolute numbers per cell type (right). (C) UMAP of 41,284 cells based on OSCC scRNAseq data from our cohort and colored by patients. (D) UMAP of 21,037 cells based on CD45-negative and HPV-negative primary HNSCC from Kürten et al. and colored by patients. [file 12967_2023_4102_MOESM7_ESM.pdf]

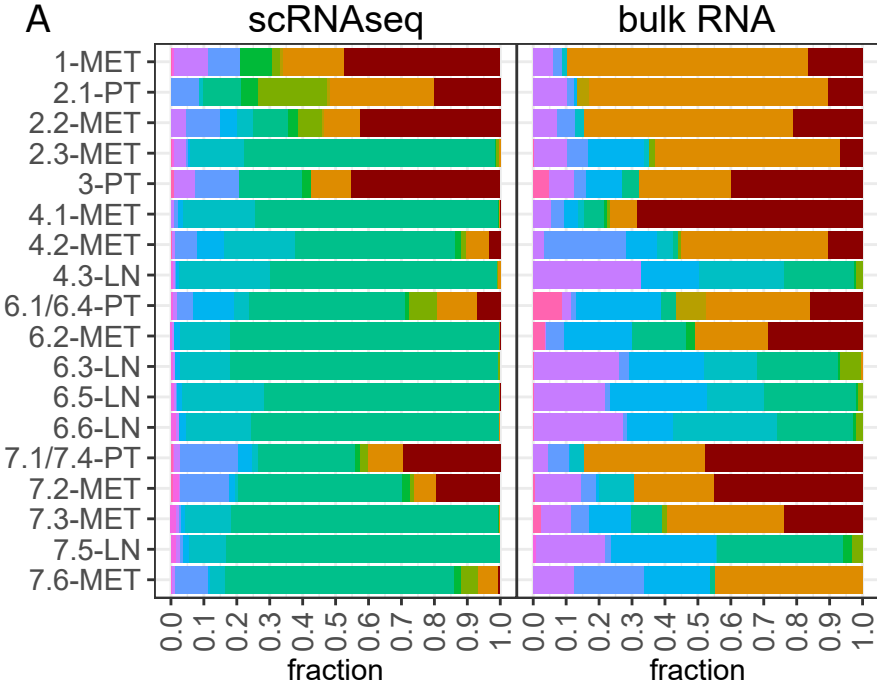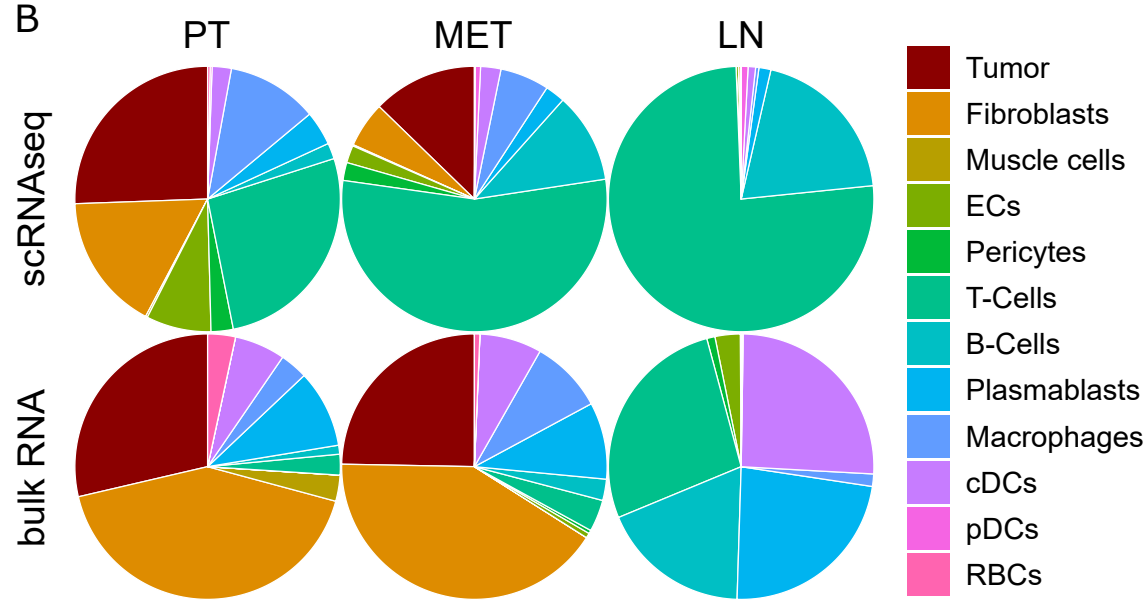

Supplement: Supplementary file 8 — Additional file 8: Figure S8. Bulk transcriptomes reveal the cellular composition of OSCC across tissue types. (A) Fractions of cell types (x-axis) across all samples (y-axis) including primary tumors (PT), metastatic lymph nodes (MET) and tumor-free lymph nodes (LN) for cells detected by scRNAseq (left panel) or deconvoluted from bulk transcriptome analysis (right panel). (B) Pie charts showing the average fraction of celltypes across samples from each tissue type, derived from scRNAseq data (top) and bulk transcriptome deconvolution (bottom) and colored by cell type. cDCs: conventional dendritic cells; pDCs: plasmacytoid dendritic cells; RBCs: red blood cells; ECs: endothelial cells. [file 12967_2023_4102_MOESM8_ESM.pdf]
