# Supplementary material for: Mesenchymal–epithelial transition in lymph node metastases of oral squamous cell carcinoma is accompanied by ZEB1 expression
Source: J Transl Med. 2023 Apr 19;21:267. doi: 10.1186/s12967-023-04102-w (PMC10114373; doi:10.1186/s12967-023-04102-w)
Supplement: Supplementary file 9 — Additional file 9: Figure S9. Characterization of OSCC-derived fibroblasts. (A) Heatmap of scaled, log-normalized expression of the top 5 differentially expressed genes (DEGs) (rows) for fibroblasts and pericytes (columns) split by their respective phenotype. DEGs are sorted from highest towards lowest log2 foldchange and row sections are ordered like column sections. (B) Normalized enrichment scores (NES) of top 5 enriched gene sets for each fibroblast phenotype. Gene sets are sorted from highest to lowest NES and the bar chart is colored by negative decadic logarithm of Benjamini-Hochberg adjusted p-values (padj). (C) Scaled, log-normalized expression of collagens (COL) (rows) across fibroblasts and pericytes split by respective phenotypes (columns). Rows are clustered by their similarity using the Euclidean distance and ward.D2 method. (D) Selected genes (y-axis) expressed across phenotypes (x-axis). Dots are colored by averaged log-normalized gene expression and dot size represents the percentage of cells expressed in this phenotype, i.e., cells with more than 1 unique molecular identifier (UMI) detected in the respective gene. (E-H) Analog to AD for fibroblasts and pericytes from Kürten et al. dataset. (I) Composition of phenotypes across tissue types in pie charts (top) and across samples as bar chart (bottom). Pie charts show the average fraction of phenotypes across fibroblasts and pericytes for each tissue type. The bar chart shows the fraction of phenotypes (x-axis) across samples (y-axis) on the left with the absolute abundance of cells on the right side, colored by tissue type. (J) Similar plot as in I for the Kürten et al. dataset. As all samples represent primary tumors, they were summarized in one pie chart. [file 12967_2023_4102_MOESM9_ESM.pdf]

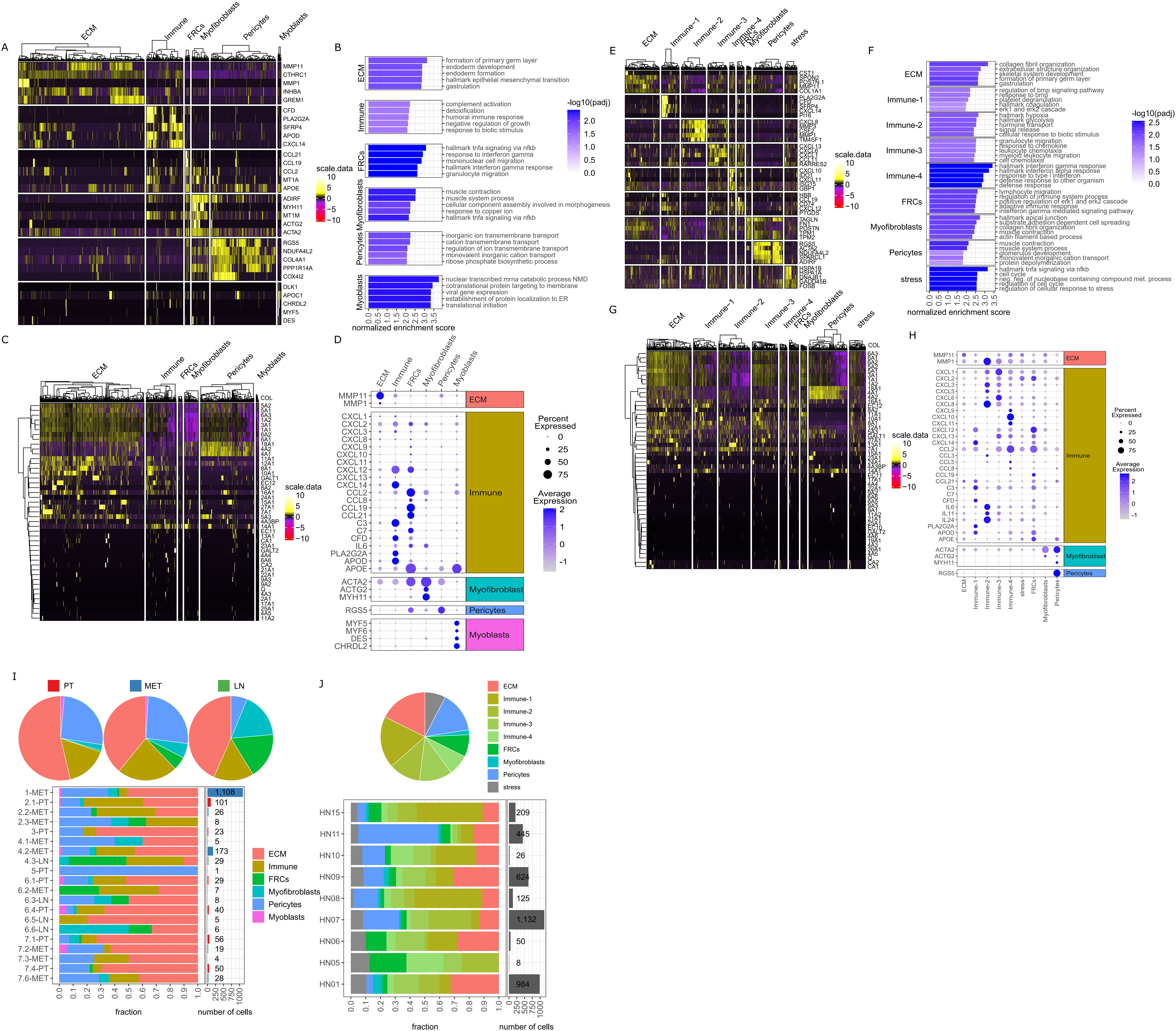

A
ECM
Immune
FRCs
Myofibroblasts
Pericytes
Myoblasts
MMP11
CTHRC1
MMP1
INHBA
GREM1
CFD
PLA2G2A
SFRP4
APOD
CXCL14
CCL21
CCL19
CCL2
MT1A
APOE
ADIRF
MYH11
MT1M
ACTG2
ACTA2
RGS5
NDUFA4L2
COL4A1
PPP1R14A
COX4I2
DLK1
APOC1
CHRDL2
MYF5
DES
scale.data
B
formation of primary germ layer
endoderm development
endoderm formation
hallmark epithelial mesenchymal transition
gastrulation
complement activation
detoxification
humoral immune response
negative regulation of growth
response to biotic stimulus
hallmark tnfa signaling via nfkb
response to interferon gamma
mononuclear cell migration
hallmark interferon gamma response
granulocyte migration
muscle contraction
muscle system process
cellular component assembly involved in morphogenesis
response to copper ion
hallmark tnfa signaling via nfkb
inorganic ion transmembrane transport
cation transmembrane transport
regulation of ion transmembrane transport
monovalent inorganic cation transport
ribose phosphate biosynthetic process
nuclear transcribed mrna catabolic process NMD
cotranslational protein targeting to membrane
viral gene expression
establishment of protein localization to ER
translational initiation
-log10(padj)
normalized enrichment score
C
COL
D
Percent Expressed
Average Expression
CXCL1
CXCL2
CXCL3
CXCL8
CXCL9
CXCL10
CXCL11
CXCL12
CXCL13
CXCL14
CCL2
CCL8
CCL19
CCL21
C3
C7
CFD
IL6
PLA2G2A
APOD
APOE
ACTA2
ACTG2
MYH11
RGS5
MYF5
MYF6
DES
CHRDL2
Myofibroblast
E
Immune−1
Immune−2
Immune−3
Immune−4
stress
CST1
SPON2
POSTN.1
COL1A1
PI16
TM4SF1
RARRES2
ISG15
GBP1
HBB
CD74
CXCL12
PTGDS
TAGLN
FN1
POSTN
TPM1
TPM2
SPARCL1
HSPA1B
HSPA1A
DNAJB1
GADD45B
FOSB
F
collagen fibril organization
extracellular structure organization
skeletal system development
formation of primary germ layer
gastrulation
regulation of bmp signaling pathway
response to bmp
platelet degranulation
hallmark coagulation
erk1 and erk2 cascade
hallmark hypoxia
hallmark glycolysis
hormone transport
signal release
cellular response to biotic stimulus
granulocyte migration
response to chemokine
leukocyte chemotaxis
myeloid leukocyte migration
cell chemotaxis
hallmark interferon gamma response
hallmark interferon alpha response
response to type i interferon
defense response to other organism
defense response
lymphocyte migration
regulation of immune system process
positive regulation of erk1 and erk2 cascade
adaptive immune response
interferon gamma mediated signaling pathway
hallmark apical junction
substrate adhesion dependent cell spreading
collagen fibril organization
muscle contraction
actin filament based process
muscle contraction
muscle system process
glomerulus development
monovalent inorganic cation transport
protein depolymerization
hallmark tnfa signaling via nfkb
cell cycle
neg. reg. of nucleobase containing compound met. process
regulation of cell cycle
regulation of cellular response to stress
G
H
CCL3
CCL5
CXCL5
CXCL6
IL11
IL24
Pericytes
I
PT
MET
LN
1-MET
2.1-PT
2.2-MET
2.3-MET
3-PT
4.1-MET
4.2-MET
4.3-LN
5-PT
6.1-PT
6.2-MET
6.3-LN
6.4-PT
6.5-LN
6.6-LN
7.1-PT
7.2-MET
7.3-MET
7.4-PT
7.6-MET
1,108
101
26
8
23
5
173
29
1
29
7
8
40
5
6
56
19
4
50
28
fraction
number of cells
J
HN15
HN11
HN10
HN09
HN08
HN07
HN06
HN05
HN01
209
445
26
624
125
1,132
50
8
984
